# The COVID-19 Pandemic and Health and Care Workers: Findings From a Systematic Review and Meta-Analysis (2020–2021)

**DOI:** 10.3389/ijph.2023.1605421

**Published:** 2023-03-03

**Authors:** Mandana Gholami, Iman Fawad, Sidra Shadan, Rashed Rowaiee, HedaietAllah Ghanem, Amar Hassan Khamis, Samuel B. Ho

**Affiliations:** ^1^ College of Medicine, Mohammed Bin Rashid University of Medicine and Health Sciences, Dubai, United Arab Emirates; ^2^ Hamdan Bin Mohammed College of Dental Medicine, Mohammed Bin Rashid University of Medicine and Health Sciences, Dubai, United Arab Emirates; ^3^ Department of Medicine, Mediclinic City Hospital, Dubai, United Arab Emirates

**Keywords:** healthcare workers, COVID-19, pandemic, COVID-19 vaccination, healthcare worker mortality

## Abstract

**Objectives:** The COVID-19 pandemic has greatly impacted health and care workers (HCW) globally, whom are considered at greater risk of infection and death. This study aims to document emerging evidence on disease prevalence, clinical outcomes, and vaccination rates of HCWs.

**Methods:** Three databases were surveyed resulting on 108 final articles between July–December 2020 (period 1) and January–June 2021 (period 2).

**Results:** Amongst the overall 980,000 HCWs identified, in period 1, the estimates were 6.1% (95% CI, 4.1–8.8) for the PCR positivity rate. Regarding outcomes, the hospitalization prevalence was 1.6% (95% CI, 0.7–3.9), and mortality rate of 0.3% (95% CI, 0.1–0.8). In period 2, the PCR positivity rate was 8.1% (95% CI, 4.6–13.8). Analysis of outcomes revealed a hospitalization rate of 0.7% (95% CI 0.3–1.8), and average mortality rate of 0.3% (95% CI 0.1–0.9). Our analysis indicated a HCW vaccination rate of 59.0% (95% CI, 39.4–76.1).

**Conclusion:** Studies from the latter half of 2020 to the first half of 2021 showed a slight increasing trend in PCR positivity among HCW, along with improved clinical outcomes in the 1-year period of exposure. These results correlate well with the improving uptake of COVID-19 vaccination globally.

## Introduction

Since its emergence in late 2019, the COVID-19 pandemic had major implications on the health and wellbeing of health and care workers (HCWs) and health systems worldwide. HCWs have been and remain at the frontlines of the pandemic response and therefore, the prevalence and subsequent implications of the COVID-19 infections amongst HCWs has become an area of vital interest.

We previously conducted a systematic review and meta-analysis of studies published between January–June 2020, representing the “first wave” of COVID-19 around the world. We found the percentage of HCWs reported to be PCR-positive for COVID-19 to be 12.5% (95%CI 6.2–23.5), with mortality rates of 0.8% (95% CI 0.4–1.6) (1, 2). Since the reporting of these results, the COVID-19 pandemic continued to evolve affecting HCWs and the general population worldwide. The remarkably rapid development of vaccines approved for emergency use allowed the first mass vaccinations to begin in many countries as early as December 2020, with the priority given to the elderly and HCWs (3–5).

As the true magnitude of vaccination success is being realized, our goal is to shed light on the HCWs population given their occupational exposure risks, over and above their community exposure. Around the globe, different political and sociological strategies have influenced the vaccination rates amongst the HCWs and the general populations. Countries such as France, Greece, Italy, and Hungary have mandated COVID-19 vaccination for HCWs, which has markedly increased the vaccination rates amongst HCWs in these nations. This implies the need for a review of vaccination rates and efficacy in those who are at an increased risk for contracting and transmitting COVID-19 infection, such as HCWs (6).

Analysis of peer-review publications stratified by two time periods is important to partially account for the changing patterns of infections, evolving public health measures, therapeutic interventions, and the growing benefits of new vaccines. This study aims to systematically review published results related to HCWs and COVID-19 from July to December 2020 and January to June 2021, with a meta-analysis of COVID-19 infections among HCWs and the related clinical outcomes, such as mortality, hospitalizations, and ICU admissions, as well as a first look at the potential impact of the introduction of vaccinations on this population group.

## Methods

This systematic review and meta-analysis has been conducted in accordance with the Preferred Reporting Items for Systematic Reviews and Meta-Analysis (PRISMA) guidelines (7).

### Information Sources and Search Strategy

Five researchers independently searched three databases (PubMed, Scopus, Google Scholar) relative to two time periods: 1 July to 31 December 2020 and 1 January to 30 June 2021, respectively. The search was carried out from April 2021 until first week of July 2021, to ensure the inclusion of all relevant published papers until 30th June 2021. SS and IF conducted PubMed search, while RR and MG conducted search on Scopus and HG was responsible for Google Scholar search. Subsequently the data was cross-checked with the other pair to assess eligibility of the title and abstracts extracted. Any disagreements were resolved through discussion with a fourth author (SH). The search keywords were broadly grouped into four categories: “healthcare,” “COVID-19” and “miscellaneous” (Supplementary Table S1). An example of search strategy used by authors were as following: (*“Healthcare providers” OR “Healthcare personnel” OR “Healthcare workers” OR “Healthcare staff” OR “Healthcare professionals” OR “Healthcare staff” OR “Doctors” OR “Nurses” OR “Physicians” OR “Medical personnel” OR “Medics”) AND (“COVID-19” OR “SARS-COV-2” OR “Coronavirus”) AND (“Vaccination” OR “Vaccine” OR “COVID-19 Vaccine” OR “seropositivity”).* Note that the same search strategy was used for each of the two time periods, with the exception that references to vaccine or vaccination were only applicable to the second time period.

### Eligibility Criteria

The eligibility criteria were defined as follows.

(i) HCW with COVID-19 infection.
(ii) Used a cohort, case–control, nested case–control, cross-sectional, or survey study design.
(iii) Reported the risk estimation (hazard ratio HR, relative risk RR, or odds ratio OR) as well as its 95% confidence intervals (CI), or sufficient statistics to calculate them.
(iv) It is worth noting that our eligibility criteria, was not limited to a particular language. Articles other than the native language of the authors (English, Arabic) were translated using Google Translate software. This was the case for 7 articles in our study as mentioned in Supplementary Table S2.

Excluded from the study were articles reporting COVID-19 information amongst general population which did not include any HCW worker data, studies reporting on information about basic science or mechanisms of COVID-19 infection, other non-SARS-COV-2 viral infections, as well as other systematic review and meta-analyses. Only indexed journals were searched and unpublished or preprint materials were not included.

### Data Extraction and Outcome of Interest

The following items was retrieved from each published article: name of authors, date of publication, article language, study location, number of patients, study design, and outcomes in HCWs in terms of PCR result, seropositivity, vaccination rates (only applicable to studies published from January 2021 onwards), hospitalizations, ICU admissions, and mortality rate.

### Quality Assessment

NIH Quality Assessment Tool checklist was used to assess the risk of bias in all identified full-text articles (8). Twelve checklist criteria were selected, and each article was rated accordingly, with one point given for each criterion (leading to a total score range 0–12). Articles were considered as at low risk of bias (scores 9–12), moderate risk of bias (scores 6–8) and high risk of bias (sores 0–5) (see Supplementary Table S2).

### Data Analysis

Percentages and 95% confidence intervals (CI) were combined to describe the prevalence of the PCR positivity, seropositivity, vaccinations, hospitalizations, ICU admissions, and mortality rate. Meta-analysis using the random-effect model was performed to estimate the pooled prevalence and 95% CI. Pooled percentage, proportions, and corresponding 95% CI were calculated to summarize the weighted effect size for all binary variables. The measure of heterogeneity reported included the Cochran’s Q statistics, I^2^ index with the level of heterogeneity defined as poor< 25; moderate >50; and high> 75, and the tau square (T^2^) test, and the Prediction Interval (PI) which reflects the real heterogeneity of the estimation among the population where the selected studies were conducted. Publication bias was assessed using funnel plots and Egger’s test.

## Results

### Study Selection and Characteristics

**FIGURE 1:**
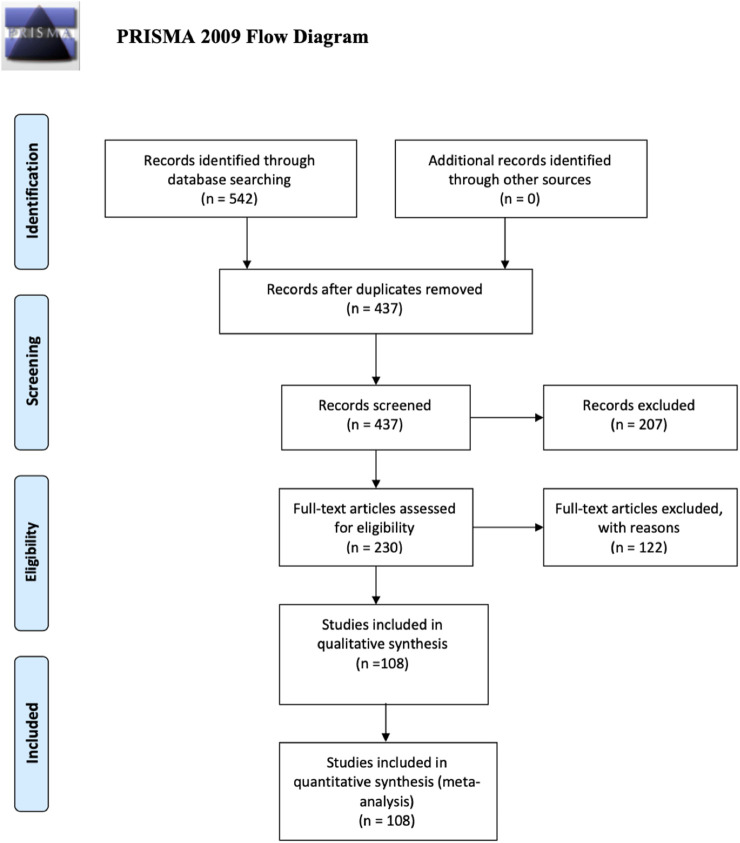
PRISMA flow diagram (UAE, 2021–2022).

**FIGURE 2:**
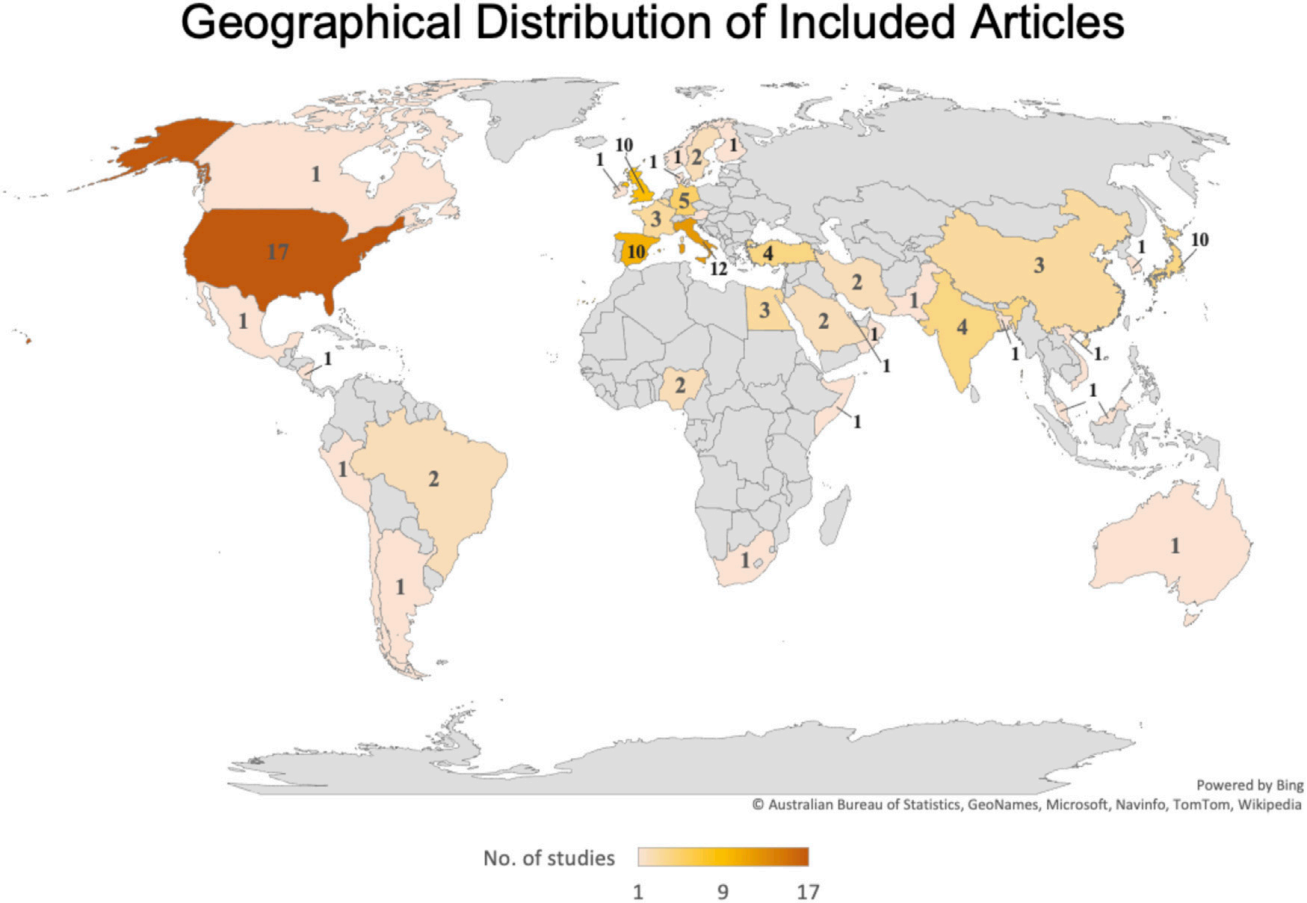
Geographical distribution of Included Articles (UAE, 2021–2022).

The systematic review retrieval flowchart is depicted in Figure 1. Three bibliographic databases (PubMed, Scopus, and Google Scholar) were searched from 1st July to 31 December 2020, and a second period of 1st January to 30 June 2021. In total, 542 studies were identified using the predefined search strategy and manual search. 107 duplicate articles were excluded and 207 studies did not meet the eligibility criteria. This resulted in 228 studies that were selected for full-text review of which 112 were excluded due to lack of information, comments, or viewpoints. The final meta-analysis included 108 studies, and the pertinent characteristics of those are shown in Supplementary Table S2. Most of those were cross-sectional studies and originated in 38 countries (Figure 2), and with the majority being published in the United States. Most of the studies showed considerable heterogeneity (I^2^ > 90%). Possible sources of this heterogeneity likely include the different settings, types of population, and methods of measurements. In period 1 no evidence of publication bias as demonstrated by Egger’s test (P-value >0.05) except for estimation of the PCR positivity (Table 1 and Supplementary Figure S1B). In period 2 there was evidence of potential bias by Egger’s test for ICU and mortality, as could be expected with a limited number of results studies with divergent (Table 2). Note that the parameters estimation of the variables in the included studies were based on proportions and 95% CIs. As indicated in Supplementary Table S3, six variables were extracted and included in the meta-analysis.

**TABLE 1:** Meta-Analysis outcomes for COVID-19 amongst healthcare workers Period 1: July to December 2020 (UAE, 2021–2022).

Item	No. of studies	Prevalence%	95% CI	n-1	Q	I^2^	T^2^	P-value	Egger’s test P
Co-morbidities and Outcomes for HCW in reported overall population									
Co-morbidities	15	19.9	13.6–28.1	14	6064.70	99.77	0.76	<0.001	0.4001
PCR positivity	44	6.1	4.1–8.8	41	21218	99.80	1.78	<0.001	0.0205
Seropositivity	27	7.9	5.5–11.2	26	4237.51	99.39	0.94	<0.001	0.8842
Hospitalization	20	1.6	0.70–3.90	19	8283.77	99.77	4.13	<0.001	0.1361
ICU	12	0.4	0.0–3.6	11	3009.74	99.64	15.29	<0.001	0.6151
Mortality	18	0.3	0.08–0.77	17	874.64	98.06	5.08	<0.001	0.0645
Outcomes for PCR -positive HCW									
Hospitalization	18	13.6	9.0–20.1	17	1418.62	98.73	0.96	<0.001	0.1621
ICU	12	3.3	1.2–8.8	11	603.84	98.18	3.13	<0.001	0.3239
Mortality	18	1.3	0.8–2.2	17	136.20	87.52	0.71	<0.001	0.3655

Q Cochran’s Q statistic for heterogeneity.

I^2^ Index for the degree of heterogeneity.

T^2^ Tau-squared measure of heterogeneity.

P-value for heterogeneity (Not significant for small studies).

Egger’s test P for bias (Not significant then no bias).

**TABLE 2:** Meta-Analysis outcomes for COVID-19 amongst healthcare workers Period 2: January to June 2021 (UAE, 2021–2022).

Item	No. of studies	Prevalence%	95% CI		n-1	Q	I^2^	T^2^	P-value	Egger’s test P
Co-morbidities and Outcomes for HCW in reported overall population										
Co-morbidities	14	28.6	23.9–33.8		13	592.15	97.81	0.19	<0.001	0.6116
PCR positivity	20	8.1	4.6–13.8		19	2182.82	99.13	1.79	<0.001	0.4448
Seropositivity	34	7.4	5.3–10.3		33	5469.8	99.4	1.0	<0.001	0.2425
Hospitalization	8	0.7	0.3–1.8		8	51.96	86.53	1.18	<0.001	0.9711
ICU	6	0.2	0.0–2.8		5	113.55	95.60	9.30	<0.001	0.0167
Mortality	5	0.3	0.1–0.9		4	5.988	33.19	0.455	0.200	0.0203
Vaccination	7	59.0	39.4–76.1		7	9921.39	99.5	1.14	<0.001	0.4159
Outcomes for PCR -positive HCW										
Hospitalization	8	2.0	1.0–4.2		7	33.73	79.25	0.72	<0.001	0.05560
ICU	6	0.7	0.1–4.1		5	55.31	90.96	4.34	<0.001	0.0067
Mortality	5	0.6	0.30–1.2		4	3.58	000	000	0.466	0.0592

Q Cochran’s Q statistic for heterogeneity.

I^2^ Index for the degree of heterogeneity.

T^2^ Tau-squared measure of heterogeneity.

P-value for heterogeneity (Not significant for small studies).

Egger’s test P for bias (Not significant then no bias).

### HCW Infections and Outcomes

The total number of HCWs analyzed in this meta-analysis was 980,296 (including 898,203 (91.6%) from period 1 and 82,093 (8.4%) from period 2). The main outcomes of interest (PCR positivity, antibody seropositivity, hospitalizations, ICU admissions, mortality, and vaccination rates) for the two time periods are presented in Tables 1, 2, respectively. For these outcomes articles that did not include a denominator from which the sample was derived were excluded, (i.e., the size of the subject population from which the HCWs sample was taken).

### Period 1 (July–December 2020)

During period 1, the estimated PCR positivity rate among 44 studies was 6.1% (95% CI, 4.1–8.8) (Table 1). In relative terms, the prevalence of seropositivity was found to be 7.90% (95% CI, 5.5–11.2) among 27 studies. With regards to HCWs outcomes, the hospitalization prevalence was 1.6% (95% CI, 0.7–3.9) among 20 articles analyzed, and ICU admission prevalence 0.4% (95% CI, 0–3.6) among 12 studies, respectively. Our analysis across 18 studies estimated an average mortality rate of 0.3% (95% CI, 0.10–0.80) among HCWs (Supplementary Figure S1).

In addition, an analysis of outcomes among only PCR positive HCWs was performed. The hospitalization rate of PCR positive HCWs was 13.6% (95% CI, 9.0–20.1), the ICU admission rate was 3.3% (95% CI, 1.2–8.8), and an average mortality rate was 1.3% (95% CI, 0.8–2.2) (Table 1).

### Period 2 (January–June 2021)

For the first half of the year 2021, our analysis pooled estimate of PCR positivity among HCWs using 20 studies was 8.1% (95% CI, 4.6–13.8) (Table 2). The prevalence of seropositivity was 7.4% (95% CI, 5.3–10.3) estimated from 34 studies. The hospitalization rate was 0.7% (95% CI 0.3–1.8) estimated from 8 studies, the ICU admission rate was 0.2% (95% CI, 0.0–2.80) estimated from 6 studies, and an average mortality rate of 0.3% (95% CI 0.1–0.9) estimated from 5 studies, respectively (Supplementary Figure S2). Special to this period is the availability of data related to COVID-19 vaccination rates among HCWs estimated to be 59.0% (95 CI, 39.4–76.1) using results from 7 studies. Overall comorbidities were present in 25.0% of the HCW (95% CI, 24.4–32.5).

A sensitivity analysis for outcomes involving only PCR positive HCWs revealed an average hospitalization rate of 2.0% (95% CI 1.0–4.2), an ICU admission rate of 0.7% (95% CI 0.1–4.1), and a mortality rate of 0.6% (95% CI 0.3–1.2), respectively (Table 2).

### An Analysis of HCWs Outcomes by Geographical Regions

An analysis of PCR positivity and mortality rates grouped by geographical regions was also performed (Supplementary Figure S2H). In period 1, the highest average PCR positivity rate was estimated for the African region (18.29%, 95% CI 12.40–26.15). In contrast, the highest mortality rate was estimated for the Western Pacific Region (2.4%; 95% CI, 0.6–9.09).

In period 2, the meta-analysis indicates that the Eastern Mediterranean Region exhibited the highest PCR positivity rate of 67.35% (95% CI, 12.40–96.78). In contrast, the highest average mortality rate was estimated to be in the African region with 0.64% (95% CI, 0.16–2.53). Due to the paucity of data, it is difficult to further compare changes in these outcomes over time by geographical region. For example, data related to PCR positivity from Africa was only available in period 1 and included only 3 studies with a total HCW population of 1003.

### A Comparison of HCWs Outcomes Over Time

**FIGURE 3:**
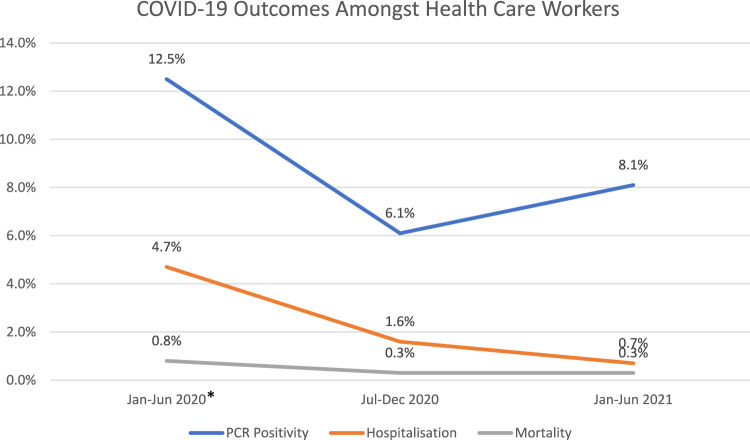
Outcome Trends during three 6-month time periods since the beginning of the pandemic (UAE, 2021–2022). *Note: Data displayed from January–June 2020 are derived from our previous publication [ref (1, 2)].

We compared our current results with our previous ones related to the first half of 2020 (1, 2). Results illustrate a decreasing trend of HCWs hospitalizations, and mortality rates over the three 6-month time periods (Figure 3). The trend of HCWs PCR positivity is slightly increased for the period January–June 2021, which indicates a slight evolution of the pandemic and the coincidence with the emergence of variants.

## Discussion

This analysis brought together a total of 108 articles pertaining to two periods, July–December 2020 and January–June 2021, to study the trends of PCR positivity rate, seropositivity, vaccination rate, hospitalization, ICU admission and mortality amongst HCWs. Compared with our previously published analysis of the period of January-June 2020, estimates indicate a consistent attenuated trend of hospitalizations and mortality rate through the subsequent three 6-month periods, with some indication that the mortality rate remaining constant between July-December 2020 and January-June 2021 (Figure 3). Conversely, PCR positivity among HCWs indicated a reasonable decline in the period July to December 2020 followed by a slight recovery in the period January–June 2021 (Figure 3). Because the global uptake of COVID-19 vaccinations only started in the beginning of 2021, any attributed (or anticipated) reductions in mortality due to vaccination protection may become detectable for documentation in time periods beyond June 2021.

Despite the paucity of data by geography and by time period, our analysis touched on some regional differentials. Our results indicate that between July and December 2020, the African region depicted the highest estimated PCR positivity rate, while in the following months of January-June 2021, the highest estimated PCR positivity rate belonged to the Eastern Mediterranean region. With regards to the mortality rate, the Western Pacific region exhibited the highest mortality rate during July-December 2020, and in the following months of January-June 2021, the position shifted to be that of the African region. These changing trends in PCR positivity and mortality rates could be indicative of the pandemic waves of progression globally. Note that there were potentially significant measures of bias noted in the PCR positivity data and data presented for the first period. This is exemplified by the Funnel plot of the skewness of the standard errors of the data (Supplementary Figure S1B). These measures of bias do not affect the result but it tells us about the conflicting outcomes of the studies in the meta analysis, and is exploratory more than explanatory. Note also that potentially significant bias was shown for ICU and mortality data in the second period, due to the very small number of studies with conflicting outcomes available.

According to WHO surveillance statistics, COVID-19 had caused 3.45 million fatalities between January 2020 and May 2021. Of the millions of fatalities, only 6,643 HCWs deaths had been reported to the WHO as of 16 May 2021. Several data source and analyses–including peer-reviewed articles, reports from governments and healthcare professional organizations, press and media coverage across the countries–contradicts this count which a appears to significantly underreport the global death toll of HCWs due to COVID-19 (9). According to COVID-19 surveillance data reported to WHO by its member states, HCWs had more than three times the risk of infection (10) specifically in the earlier months of the pandemic. In contrast, there were severe gaps in reporting equivalent levels of mortality.

Population-based estimates of COVID-19-related deaths in HCWs was estimated to be approximately 115,493 out of an estimated global total of 135 million HCWs in the workforce (9). This population-based estimate however is likely to be an underestimate in its own right due to marked under-reporting in the overall number of COVID-19 deaths, especially from Africa, South-East Asia, Eastern Mediterranean and the Western Pacific regions (9). Notwithstanding that given the two periods of our study, the highest PCR positivity rates were found in the African and Eastern Mediterranean regions, respectively, and the highest mortality rates in the Western Pacific and African regions, respectively.

There could be many reasons to explain the differences between estimated and actual reported numbers of COVID-19 related infections and deaths. These include variable limitations in the capacity for testing and monitoring COVID-19 infections in HCWs across countries, under or no reporting of COVID-19 deaths occurring outside of hospitals or other healthcare settings, reluctance in some countries to disclose deaths from COVID-19 in healthcare workers, lack of testing of mild and asymptomatic cases, and false negative or false positive tests due to limitations in the specific test and testing conditions (9, 11–13). As a result, many untested HCWs deaths may have been excluded from total mortality figures.

Important to note that the wide range predictions of future infection scenarios obtained from infectious disease models early in the pandemic were likely due to the under-detection and delayed reporting of RT-PCR confirmed COVID-19 cases (11, 14). We found that there was a marked shift in the focus of articles from the analysis of PCR positivity rate amongst HCWs, to the seropositivity overtime, highlighting the potential importance of seropositivity in estimating untested/unreported infections. Although the results were quite heterogenous between studies and settings, they consistently showed that the true number of people who have been infected is higher than the official number of tested and confirmed cases. One such example can be seen from the Prevalence of Antibodies to SARS-CoV-2 in the Irish Healthcare Workers (PRECISE) study–aimed at estimating seroprevalence among HCWs in two Irish hospitals between October 2020 and March/April 2021. Despite high rates of testing for active infection among these HCWs, the study estimated that 39% of HCWs with detectable antibodies had not received a positive RT-PCR diagnosis (15). In another shorter time-span study from a large Swedish emergency care hospital, 3,981 HCWs provided serum samples and questionnaire information between May to June 2020. At the start of the study, the total seroprevalence was 18% and increased with the duration of the study. One-fifth of the seropositive HCWs were completely asymptomatic and the odds for seropositivity were higher among those who worked with COVID-19 patients (16).

In a meta-analysis conducted by Galanis et al, including 49 studies with 127,480 HCWs from inception of seroprevalence testing to 24th August 2020, the estimated overall seroprevalence of SARS-CoV-2 antibodies among HCWs was 8.7% (17). The results of this meta-analysis are similar to our highest average seropositivity rate of 7.9% within the period of July–December 2020, as well as 8.24% within the period of January–June 2021. The highest seroprevalence in the aforementioned meta-analysis was observed amongst studies conducted in North America (12.7%) compared with those conducted in Europe (8.5%), Africa (8.2%) and Asia (4%). Overall, there was little difference in seropositivity between the two time periods. Different study populations, different antibody tests with varying sensitivity and specificity, varied study designs, different lockdown and quarantine protocols, and different data collection dates may have all contribute to differences in seroprevalence within studies across the geographical locations and time periods (17).

Vaccination against COVID is critical in controlling the pandemic, especially amongst HCWs as they are and have been the in frontline since the beginning. In our analysis, vaccination data was only applicable to the period of January–June 2021. The first vaccine to be approved by the World Health Organization (WHO) was BNT16b2/COMIRNATY Tozinameran (INN), commonly known as Pfizer BioNtech, in December 2020. Since then, a total of 8 vaccines have been approved for use by the WHO around the world, with many others in clinical trials (18). Limited data exists in the literature reporting vaccination rate amongst HCWs during the early time periods of the pandemic. In a study conducted by Narayan et al., to determine the vaccination among HCWs, vaccination data from a total of 14,837 healthcare personnel from 20 different hospitals in India were prospectively analyzed. It was determined that the vaccination was taken up by 13,335 HCWs in total (90%). The infection rate in vaccinated HCWs was 710 (6.04%), which was considerably lower than the infection rate in unvaccinated HCWs, which was 148 (9.9%). ICU admissions were also lower in vaccinated compared with unvaccinated HCWs, but there was no mortality in either group in this study (19). Since completing the analysis of our study, many studies have emerged, further asserting the efficacy of vaccination amongst HCWs and improved health outcomes (20–23). As of March 2022, over 11 billion vaccination doses have reportedly been distributed worldwide and according to WHO COVID-19 vaccination dashboard, more than 70% of HCWs had been fully vaccinated (24, 25).

Our study poses several limitations. First, there was a major difference in the number of extracted “articles” in the first and second “periods”, reflecting the possible loss of focus on epidemiology of HCW with COVID-19. Second, majority of our articles were from the US. Ever since our previous publication which evaluated HCW outcomes with COVID-19 over the first 6 months of the pandemic, the literature has faced a major shift from publications from China to the US, with a possible explanation of the sudden sharp increase in number of COVID-19 infections amongst the US population. Third, we included only published and indexed papers in the analysis, excluding potential data from other sources and registries. Last, with different COVID-19 guidelines implemented over different parts of the world, it is expected that different diagnostic tests are used for COVID-19 detection, particularly when it comes to serological testing; and different outcomes may be expected due to differences in treatments available.

As we conclude this study, the world continues to witness new phases of the COVID-19 pandemic with new waves of old and new variants alongside reasonable progress in vaccination rates. The race continues between evolving variants and changing natural and vaccine induced immunity of the global population. Therefore, it remains crucially important to monitor the situation of HCWs infections and related outcomes in tandem with ongoing research into the efficacy of protective vaccination and of infection control practices. These data would enable policymakers and the scientific community to better understand how the virus took hold and evolves in different settings and populations. It would also facilitate a retrospective evaluation of the reliability of infectious disease modelling that can better inform public health planning and preparedness for future health emergencies (26).
