# Supplementary material for: The COVID-19 Pandemic and Health and Care Workers: Findings From a Systematic Review and Meta-Analysis (2020–2021)
Source: Int J Public Health. 2023 Mar 3;68:1605421. doi: 10.3389/ijph.2023.1605421 (PMC10020210; doi:10.3389/ijph.2023.1605421)
Supplement: Supplementary file 1 [file DataSheet1.docx]

**Supplementary Materials**

Table of Contents

[Supplementary Table 1: Keywords (UAE, 2020-2021) 3](#_Toc127896862)

[Supplementary Table 2: Characteristics of articles included in this study (UAE, 2020-2021) 4](#_Toc127896863)

[Supplementary Table 3: Table of outcome data (UAE, 2020-2021) 14](#_Toc127896864)

[Supplementary Figure 1. Forest Plots of Outcomes amongst Total Healthcare Workers Period 1: July-December 2020 23](#_Toc127896865)

[A. Co-morbidities (UAE, 2020-2021) 23](#_Toc127896866)

[B. PCR Positivity Forest Plot and Funnel Plot (UAE, 2020-2021) 24](#_Toc127896867)

[C. Seropositivity (UAE, 2020-2021) 26](#_Toc127896868)

[D. Hospitalizations (UAE, 2020-2021) 27](#_Toc127896869)

[E. ICU Admission (UAE, 2020-2021) 28](#_Toc127896870)

[F. Mortality (UAE, 2020-2021) 29](#_Toc127896871)

[Supplementary Figure 2. Forest Plots of Outcomes Amongst Total Healthcare Workers Time Period 2: January-June 2021 30](#_Toc127896872)

[A. Co-morbidities (UAE, 2020-2021) 30](#_Toc127896873)

[B. PCR positivity (UAE, 2020-2021) 31](#_Toc127896874)

[C. Seropositivity (UAE, 2020-2021) 32](#_Toc127896875)

[D. Hospitalizations (UAE, 2020-2021) 33](#_Toc127896876)

[E. ICU admissions (UAE, 2020-2021) 34](#_Toc127896877)

[G. Vaccinations (UAE, 2020-2021) 36](#_Toc127896878)

[H. Global comparison – PCR positivity (UAE, 2020-2021) 37](#_Toc127896879)

[References 39](#_Toc127896880)

# **Supplementary Table 1: Keywords (UAE, 2020-2021)**

| Healthcare | COVID-19 | Misc. |
| --- | --- | --- |
| Doctors | COVID-19 | Vaccine |
| Physicians | SARS-COV-2 | Seropositivity |
| Nurses | Coronavirus |  |
| Healthcare providers | nCOV |  |
| Healthcare workers | SARS-COV-2 |  |
| Healthcare professionals | Pandemic |  |
| Healthcare personnel | Infection |  |
| Healthcare Staff |  |  |
| Front-liners |  |  |
| Medical personnel |  |  |
| Medics |  |  |

# **Supplementary Table 2: Characteristics of articles included in this study (UAE, 2020-2021)**

| # | Author | Title | Publica-tion Date | Location | Language | Study Design | Journal | Quality Score | Total # HCW | References |
| --- | --- | --- | --- | --- | --- | --- | --- | --- | --- | --- |
| 1 | Fafi-Kremer et al. | Serologic responses to SARS-CoV-2 infection among hospital staff with mild disease in eastern France. | July-20 | France | English | Cross-sectional | EBioMedicine | 8 | 160 | (1) |
| 2 | Zheng et al. | Characteristics and transmission dynamics of COVID-19 in healthcare workers at a London teaching hospital | July-20 | United Kingdom | English | Cross-sectional | J Hosp Infect | 9 | 958 | (2) |
| 3 | Lombardi et al. | Characteristics of 1573 healthcare workers who underwent nasopharyngeal swab testing for SARS-CoV-2 in Milan, Lombardy, Italy | July-20 | Italy | English | Observational Cohort | Clin Microbiol Infect | 8 | 1573 | (3) |
| 4 | Epstude et al. | Seroprevalence of COVID-19 antibodies in the cleaning and oncological staff of a municipal clinic | July-20 | Germany | German/English | Cross-sectional | GMS Hyg Infect Control | 8 | 65 | (4) |
| 5 | Folgueira et al. | SARS-CoV-2 infection in health care workers in a large public hospital in Madrid, Spain, during March 2020. | July-20 | Spain | English | Retrospective cohort study | J Hosp Infect | 6 | 1911 | (5) |
| 6 | Sotgiu et al. | SARS-CoV-2 specific serological pattern in healthcare workers of an Italian COVID-19 forefront hospital | July-20 | Italy | English | Cross-sectional | BMC Pulm Med | 7 | 202 | (6) |
| 7 | Bampoe et. al. | A Cross-sectional study of immune seroconversion to SARS-CoV-2 in frontline maternity health professionals | August-20 | United Kingdom | English | Cross-sectional | Anaesthesia | 6 | 200 | (7) |
| 8 | Fill Malfertheiner et al. | Immune response to SARS-CoV-2 in health care workers following a COVID-19 outbreak: A prospective longitudinal study. | August-20 | Germany | English | Prospective longitudinal Cross-sectional | J Clin Virol | 7 | 166 | (8) |
| 9 | Fuereder et al. | SARS-CoV-2 seroprevalence in oncology healthcare professionals and patients with cancer at a tertiary care centre during the COVID-19 pandemic. | August-20 | Austria | English | Mixed retrospective/prospective cohort | ESMO Open | 7 | 62 | (9) |
| 10 | Garcia-Basteiro et al. | Seroprevalence of antibodies against SARS-CoV-2 among health care workers in a large Spanish reference hospital | August-20 | Spain | English | Cross-sectional | Nat Commun | 11 | 578 | (10) |
| 11 | Harada et al. | Control of a Nosocomial Outbreak of COVID-19 in a University Hospital | August-20 | Japan | English | Observational Cohort | Open Forum Infect Dis | 4 | 697 | (11) |
| 12 | Hartmann et al. | Coronavirus 2019 (COVID-19) Infections Among Healthcare Workers, Los Angeles County, February - May 2020 | August-20 | United States | English | Cross-sectional | Clin Infect Dis | 6 | 5118 | (12) |
| 13 | Iversen et al. | Risk of COVID-19 in health-care workers in Denmark: an observational cohort study | August-20 | Denmark | English | Observational Cohort | Lancet Infect Dis | 6 | 28,792 | (13) |
| 14 | Jeremias et al. | Prevalence of SARS-CoV-2 Infection Among Health Care Workers in a Tertiary Community Hospital | August-20 | United States | English | Cross-sectional | JAMA Intern Med | 5 | 3046 | (14) |
| 15 | Vilela-Estrada et al. | Clinical manifestations and course of the first six reported cases of COVID-19 in the medical doctors of Peru | August-20 | Peru | Spanish | Case report | Medwave | *2* | *6* | *(15)* |
| 16 | Lackermair et al. | Infection with SARS-CoV-2 in primary care health care workers assessed by antibody testing | August-20 | Germany | English | Observational Cohort | Fam Pract | 6 | 151 | (16) |
| 17 | Malik et al. | Clinical presentation, management and in-hospital outcome of healthcare personnel with COVID-19 disease | August-20 | Bangladesh | English | Cross-sectional | Cureus | 5 | 1409 | (17) |
| 18 | Plebani et al. | SARS-CoV-2 serosurvey in health care workers of the Vento region | August-20 | Italy | English | Cross-sectional | Clin Chem Lab Med | 8 | 8,285 | (18) |
| 19 | Rizza et al. | High body mass index and night shift work are associated with COVID-19 in health care workers | August-20 | Italy | English | Cross-sectional | J Endocrinol Invest | 6 | 1180 | (19) |
| 20 | Stock et al. | COVID-19 Infection Among Healthcare Workers: Serological Findings Supporting Routine Testing | August-20 | United States | English | Cross-sectional | Front Med | 9 | 98 | (20) |
| 21 | Dacosta-Urbieta et al. | Seroprevalence of SARS-CoV-2 Among Pediatric Healthcare Workers in Spain | September-20 | Spain | English | Cross-sectional | Frontiers in Pediatr | 11 | 175 | (21) |
| 22 | García-Sierra et al. | Descriptive study of the health service workers of a Primary Care Department confined by Covid-19. | September-20 | Spain | Spanish | Cross-sectional | Rev Esp Salud Publica | 10 | 1418 | (22) |
| 23 | Guerrero-Torres et al. | Clinical Characteristics and Mortality of Healthcare Workers with SARS-CoV-2 infection in Mexico City | September-20 | Mexico | English | Cross-sectional | Clin Infect Dis | 11 | 16,446 | (23) |
| 24 | Kassem et al. | SARS-CoV-2 infection among healthcare workers of a gastroenterological service in a tertiary care facility | September-20 | Egypt | English | Cross-sectional | Arab J Gastroenterol | 10 | 74 | (24) |
| 25 | Self et al. | Seroprevalence of SARS-CoV-2 Among Frontline Health Care Personnel in a Multistate Hospital Network - 13 Academic Medical Centers, April-June 2020. | September-20 | United States | English | Cross-sectional | MMWR Morb Mortal Wkly Rep | 9 | 3248 | (25) |
| 26 | Vandercam et el. | Clinical characteristics and humoral immune response in healthcare workers with COVID-19 in a teaching hospital in Belgium | September-20 | Belgium | English | Retrospective study | J Hosp Infect | 9 | 7757 | (26) |
| 27 | Zhang et al. | Factors associated with asymptomatic infection in health-care workers with severe acute respiratory syndrome coronavirus 2 infection in Wuhan, China: a multicentre retrospective cohort study | September-20 | China | English | Retrospective cohort | Clin Microbiol Infect | 11 | 8553 | (27) |
| 28 | Alajmi et al. | COVID-19 infection among healthcare workers in a national healthcare system: The Qatar experience | September-20 | Qatar | English | Cross-sectional | Int J Infect Dis | 9 | 16912 | (28) |
| 29 | Misra-Hebert et al. | Impact of the COVID-19 pandemic on healthcare workers' risk of infection and outcomes in a large, integrated health system | September-20 | United States | English | Retrospective cohort study | J Gen Intern Med | 11 | 6145 | (29) |
| 30 | Moscola et al. | Prevalence of SARS-CoV-2 antibodies in health care personnel in the New York City area | September-20 | United States | English | Cohort study | JAMA | 11 | 40,329 | (30) |
| 31 | Shah et al. | Risk of hospital admission with coronavirus disease 2019 in healthcare workers and their households: nationwide linkage cohort study | September-20 | United Kingdom | English | Cross-sectional | BMJ | 11 | 158,445 | (31) |
| 32 | Shields et al. | SARS-CoV-2 seroprevalence and asymptomatic viral carriage in healthcare workers: a Cross-sectional study | September-20 | United Kingdom | English | Cross-sectional | Thorax | 10 | 516 | (32) |
| 33 | Meng et al. | Epidemiological investigation of OHCWs with COVID-19 | September-20 | China | English | Retrospective cohort study | J of Dental Res | 7 | 31 | (33) |
| 34 | Perotti et al. | SARS-CoV-2 antibody prevalence in health care workers of Lodi hospital, the COVID-19 Italian Epicentre | September-20 | Italy | English | Cross-sectional | SSRN Electronic Journal | 8 | 2415 | (34) |
| 35 | Fell et al. | SARS-CoV-2 Exposure and Infection Among Health Care Personnel - Minnesota, March 6-July 11, 2020. | October-20 | United States | English | Prospective cohort | MMWR Morb Mortal Wkly Rep | 9 | 5,374 | (35) |
| 36 | Ibrahim et al. | COVID-19 in health-care workers: Testing and outcomes at a Victorian tertiary children's hospital | October-20 | Australia | English | Observational Cohort | J Paediatr Child Health | 7 | 1,964 | (36) |
| 37 | Kambhampati et al. | COVID-19-Associated Hospitalizations Among Health Care Personnel - COVID-NET, 13 States, March 1-May 31, 2020 | October-20 | United States | English | Cross-sectional | MMWR Morb Mortal Wkly Rep | 10 | 438 | (37) |
| 38 | Rudberg et al. | SARS-CoV-2 exposure, symptoms and seroprevalence in healthcare workers in Sweden | October-20 | Sweden | English | Cross-sectional | Nat Commun | 8 | 2149 | (38, 39) |
| 39 | Akyala et al. | Severe Acute Respiratory Syndrome Coronavirus 2 (SARS-CoV-2) infection among health care workers in Nasarawa State, Nigeria: implications for infection prevention and control measures | October-20 | Nigeria | English | Case series analysis | Pan Afr Med J | 7 | 648 | (39) |
| 40 | Augusto et al. | Healthcare Workers Bioresource: Study outline and baseline characteristics of a prospective healthcare worker cohort to study immune protection and pathogenesis in COVID-19 | October-20 | United Kingdom | English | Prospective cohort | Wellcome Open Res | 11 | 400 | (40) |
| 41 | Barrett et al. | Prevalence of SARS-CoV-2 infection in previously undiagnosed health care workers in New Jersey, at the onset of the U.S. COVID-19 pandemic | November-20 | United States | English | Prospective cohort | BMC Infect Dis | 9 | 546 | (41) |
| 42 | Brant-Zawadzski et al. | SARS-CoV-2 antibody prevalence in health care workers: Preliminary report of a single center study | November-20 | United States | English | Cross-sectional | PLoS ONE | 6 | 2924 | (42) |
| 43 | Goenka et al. | Seroprevalence of COVID-19 Amongst Health Care Workers in a Tertiary Care Hospital of a Metropolitan City from India. | November-20 | India | English | Cross-sectional | J Assoc Physicians India | 10 | 1122 | (43) |
| 44 | Gras-Valenti et al. | Sero-epidemiological study of SARS-CoV-2 infection among healthcare personnel in a healthcare department | November-20 | Spain | Spanish | Cross-sectional | Enferm infecc Microbiol Clin | 10 | 4179 | (44) |
| 45 | Ko et al. | Serologic Evaluation of Healthcare Workers Caring for COVID-19 Patients in the Republic of Korea | November-20 | South Korea | English | Cross-sectional | Front Microbiol | 9 | 432 | (45) |
| 46 | Eren et al. | Evaluation of health care workers with COVID-19. | November-20 | Turkey | Turkish | Retrospective Cross-sectional | Klimik Derg. | 7 | 1325 | (46) |
| 47 | Mahajan et al. | Prevalence and clinical presentation of COVID-19 among healthcare workers at a dedicated hospital in India | November-20 | India | English | Observational retrospective monocentric study | J Assoc Physicians India | 8 | 3711 | (47) |
| 48 | Martin et al. | Demographic and occupational determinants of anti-SARS-CoV-2 IgG seropositivity in hospital staff | November-20 | United Kingdom | English | Case-series | J Public Health (Oxf) | 10 | 10662 | (48) |
| 49 | Rubbi et al. | Healthcare personnel exposure to COVID - 19: an observational study on quarantined positive workers | November-20 | Italy | English | Retrospective observational study | Acta Biomedica | 6 | 3565 | (49) |
| 50 | Abohamr et al. | Clinical characteristics and in-hospital outcome of medical staff infected with COVID-19 in Saudi Arabia | December-20 | Saudi Arabia | English | Retrospective Cross-sectional | Saudi Med J. | 8 | 108 | (50) |
| 51 | Barallat et al. | Seroprevalence of SARS-CoV-2 IgG specific antibodies among healthcare workers in the Northern Metropolitan Area of Barcelona, Spain, after the first pandemic wave | December-20 | Spain | English | Prospective Cross-sectional | PLoS ONE | 11 | 7563 | (51) |
| 52 | Buonafire et al. | High prevalence of SARS-CoV-2 infection among symptomatic healthcare workers in a large university tertiary hospital in São Paulo, Brazil | December-20 | Brazil | English | Cross-sectional | BMC Infect Dis | 10 | 295 | (52) |
| 53 | Foo et al. | COVID-19 testing strategy in response to infection among healthcare workers in a large non-COVID-designated hospital. | December-20 | Malaysia | English | Cross-sectional | Hosp Pract | 7 | 3336 | (53) |
| 54 | Fujita et al. | Quantitative SARS-CoV-2 Antibody Screening of Healthcare Workers in the Southern Part of Kyoto City During the COVID-19 Pre-pandemic Period | December-20 | Japan | English | Cross-sectional | Front Public Health | 8 | 92 | (54) |
| 55 | Galán et al. | Hospital-Wide SARS-CoV-2 seroprevalence in health care workers in a Spanish teaching hospital | December-20 | Spain | English | Cross-sectional | Enferm Infecc Microbiol Clin (Engl Ed) | 9 | 2590 | (55) |
| 56 | Insúa et al. | Seroprevalence of SARS-CoV-2 antibodies among physicians from a children's hospital | December-20 | Argentina | English | Cross-sectional | Arch Argent Pediatr | 8 | 116 | (56) |
| 57 | Kim et al. | Comparison of COVID-19 infections among healthcare workers and non-healthcare workers | December-20 | United States | English | Cross-sectional | PLoS One | 12 | 193 | (57) |
| 58 | Krastinova et al. | Household transmission and incidence of positive SARS-CoV-2 RT-PCR in symptomatic healthcare workers, clinical course and outcome: a French hospital experience | December-20 | France | English | Cross-sectional | Occup Environ Med | 12 | 314 | (58) |
| 59 | Schwarts et al. | Epidemiology, clinical characteristics, household transmission, and lethality of severe acute respiratory syndrome coronavirus-2 infection among healthcare workers in Ontario, Canada | December-20 | Canada | English | Cross-sectional | PLOS one | 10 | 552,560 | (59) |
| 60 | Kantele et al. | SARS-CoV-2 infections among healthcare workers at Helsinki University Hospital, Finland, spring 2020: Serosurvey, symptoms and risk factors | December-20 | Finland | English | Cross-sectional | Travel Med Infect Dis | 12 | 1095 | (60) |
| 61 | Alshahrani et al. | Prevalence of the SARS-CoV-2 infection among post-quarantine healthcare workers | December-20 | Saudi Arabia | English | Prospective Cross-sectional | J Multidiscip Healthc | 10 | 301 | (61) |
| 62 | Coppeta et al. | Contact Screening for Healthcare Workers Exposed to Patients with COVID-19 | December-20 | Italy | English | Retrospective Cross-sectional | Int J Environ Res Public Health | 5 | 1006 | (62) |
| 63 | Li et al. | Mild manifestations of COVID-19 in healthcare workers | December-20 | China | English | Retrospective case series study | PLOS Negl Trop Dis | 6 | 56 | (63) |
| 64 | Mandić-Rajčević et al. | Source and symptoms of COVID-19 among hospital workers in Milan | December-20 | Italy | English | Cross-sectional | Occup Med | 8 | 185 | (64) |
| 65 | Lawati et al. | Risk of COVID-19 Infection in Healthcare Workers Exposed During Use of Non-invasive Ventilation in a Tertiary Care Hospital in Oman | December-20 | Oman | English | Prospective Cohort | Oman Med J | 5 | 38 | (64, 65) |
| 66 | Escribano Ceruelo et al. | Seroprevalence of antibodies against SARS-CoV-2 among health care workers in a pediatric monographic hospital in Madrid (Spain) | January-21 | Spain | Spanish | Prospective cohort | Enferm infecc Microbiol Clin | 8 | 1292 | (66) |
| 67 | Goldblatt et al. | Cross-sectional prevalence of SARS-CoV-2 antibodies in healthcare workers in paediatric facilities in eight countries | January-21 | Europe & South Africa | English | Cross-sectional | J Hosp Infect | 4 | 4114 | (67) |
| 68 | Gupta et al. | Seroprevalence of antiodies to SARS-CoV-2 in healthcare workers and implications of infection control practice in India | January-21 | India | English | Cross-sectional | Indian J Med Res | 8 | 3739 | (68) |
| 69 | Huete-Pérez et al. | First report on prevalence of SARS-CoV-2 infection among health-care workers in Nicaragua | January-21 | Nicaragua | English | Cross-sectional | PLOS ONE | 5 | 402 | (69) |
| 70 | Johnson et al. | Prevalence of SARS-CoV-2 antibodies among North Dakota community pharmacy personnel: A seroprevalence survey | January-21 | United States | English | Cross-sectional | J Am Pharm Assoc | 8 | 247 | (70) |
| 71 | Abdelmoniem et al | SARS-Cov-2 infection among asymptomatic healthcare workers of the emergency department in a tertiary care facility | January-21 | Egypt | English | Cross-sectional | J Clin Virol | 8 | 203 | (71) |
| 72 | Abdi et al | Preliminary findings of COVID-19 infection in health workers in Somalia: A reason for concern | January-21 | Somalia | English | Cross-sectional | Int J Infect Dis | 2 | 311 | (72) |
| 73 | Nicolas et al. | A Prospective Cohort of SARS-CoV-2-Infected Health Care Workers: Clinical Characteristics, Outcomes, and Follow-up Strategy | Januray-21 | Spain | English | Prospective Cohort | Open Forum Infect Dis | 9 | 590 | (73) |
| 74 | Olayanju et al. | SARS-CoV-2 seropositivity in asymptomatic frontline health workers in Ibadan, Nigeria | Januray-21 | Nigeria | English | Cross-sectional | Am J Trop Med Hyg | 4 | 133 | (74) |
| 75 | Olmos et al. | SARS-CoV-2 infection in asymptomatic healthcare workers at a clinic in Chile. | Janurary 21 | Chile | English | Cross-sectional | PLOS One | 9 | 413 | (75) |
| 76 | Platten et al. | Prevalence of SARS-CoV-2 in employees of a general hospital in Northrhine-Westphalia, Germany | Janurary 21 | Germany | German | Cross-sectional | Dtsch Med Wochenschr | 8 | 1363 | (76) |
| 77 | Díez-Manglano et al. | Risk of death of healthcare workers hospitalized due to COVID-19 | February-21 | Spain | English | Observational cohort | PLOS ONE | 10 | 419 | (77) |
| 78 | Comelli et al. | Serological Response to SARS-CoV-2 in Health Care Workers Employed in a Large Tertiary Hospital in Lombardy, Northern Italy | February-21 | Italy | English | Retrospective Observatioal | Microorganisms | 6 | 1893 | (78) |
| 79 | Jungo et al. | Prevalence and risk indicators of first-wave COVID-19 among oral health-care workers: A French epidemiological survey | Febuary-21 | France | English | Cross-sectional | PLOS ONE | 7 | 4172 | (79) |
| 80 | Khan et al. | SARS-CoV-2 Seroprevalence Among Healthcare Workers by Workplace Exposure Risk in Kashmir, India | Febuary-21 | India | English | Cross-sectional observational | J Hosp Med | 8 | 2915 | (80) |
| 81 | Lankarani et al. | Prevalence of Anti-SARS-CoV-2 Antibody in Hospital Staff in Double-Center Setting: A Preliminary Report of a Cohort Study From Iran | Febuary-21 | Iran | English | Cohort | Shiraz E-Med J | 10 | 494 | (81) |
| 82 | Ledda et al. | SARS-CoV-2 Seroprevalence Post-First Wave among Primary Care Physicians in Catania (Italy) | Febuary-21 | Italy | English | Cross-sectional | Trop Med Infect Dis | 9 | 133 | (82) |
| 83 | Lumley et al. | Antibody status and incidence of SARS-CoV-2 infection in health care workers. | Febuary-21 | United Kingdom | English | Prospective Cohort | NEJM | 11 | 1177 | (83) |
| 84 | Handal et al. | Comparison of SARS-CoV-2 infections in healthcare workers with high and low exposures to COVID-19 patients in a Norwegian University Hospital | Feburary-21 | Norway | English | Cross-sectional | J Infect Dis | 9 | 360 | (84) |
| 85 | Jacob et al. | Risk factors associated with SARS-CoV-2 seropositivity among US health care personnel | March-21 | United States | English | Cross-sectional | JAMA | 12 | 24749 | (85) |
| 86 | Nygren et al. | Association between SARS-CoV-2 and exposure risks in health care workers and university employees–a Cross-sectional study | Mar-21 | Sweden | English | Cross-sectional | J Infect Dis | 12 | 271 | (86) |
| 87 | Airoldi et al | High Seroprevalence of SARS-CoV-2 among Healthcare Workers in a North Italy Hospital | March-21 | Italy | English | Cross-sectional | Int J Environ Res Public Health | 10 | 2250 | (87) |
| 88 | Alkurt et al | Seroprevalence of coronavirus disease 2019 (COVID-19) among health care workers from three pandemic hospitals in Turkey | March-21 | Turkey | English | Cross-sectional | PLOS ONE | 9 | 932 | (88) |
| 89 | Johnson et al. | SARS-CoV-2 RT-PCR positivity and antibody prevalence among asymptomatic hospital-based health care workers | March-21 | United States | English | Cross-sectional | J Clin Virol | 8 | 374 | (89) |
| 90 | Mostafa et al. | SARS-CoV-2 seroconversion among 4040 Egyptian healthcare workers in 12 resource-limited healthcare facilities: A prospective cohort study. | March-21 | Egypt | English | Prospective cohort | Int J Infect Dis | 11 | 2282 | (90) |
| 91 | Oliveira et al. | SARS-Cov-2 seroprevalence and risk factors among health care workers: Estimating the risk of COVID-19 dedicated units | March-21 | Brazil | English | Prospective Cross-sectional | Am J Infect Control | 9 | 1996 | (91) |
| 92 | Özdemir at al. | SARS-CoV-2 seroprevalence among healthcare workers: Retrospective analysis of the data from a university hospital in Turkey | March-21 | Turkey | Turkish | Retrospective cohort | Mikrobiyol Bul | 7 | 774 | (92) |
| 93 | Purswani et al. | SARS-CoV-2 Seroprevalence Among Healthcare Workers by Job Function and Work Location in a New York Inner-City Hospital | March-21 | United States | English | Cross-sectional | J Hosp Med | 8 | 2749 | (93) |
| 94 | Sabetian et al. | COVID-19 infection among healthcare workers: a Cross-sectional study in southwest Iran | March-21 | Iran | English | Cross-sectional | Virol J | 6 | 273 | (94) |
| 95 | Abbas et al | Seroprevalence of SARS-CoV-2 Antibodies Among Health Care Personnel at a Health Care System in Pakistan | April-21 | Pakistan | English | Prospective Cohort | Asia Pac J | 9 | 2162 | (95, 96) |
| 96 | Colaneri et al. | The experience of the health care workers of a severely hit SARS-CoV-2 referral Hospital in Italy; incidence, clinical course and modifiable risk factor for COVID-19 infection | April-21 | Italy | English | Cross-sectional | J Public Health | 5 | 1447 | (96) |
| 97 | Kindgen-Milles et al. | Prevalence of SARS-COV-2 positivity in 516 German intensive care and emergency physicians studied by seroprevalence of antibodies National Covid Survey Germany (NAT-COV-SURV) | April-21 | Germany | English | Cross-sectional | PLOS ONE | 8 | 516 | (97) |
| 98 | Morris et al. | Prevalence of SARS-CoV-2 antibodies in pediatric healthcare workers | April-21 | United States | English | Prospective Longitudinal Cohort | Int J Infect Dis | 9 | 642 | (98) |
| 99 | Ogutlu et al. | Novel coronavirus seropositivity and related factors among healthcare workers at a university hospital during the prevaccination period: a Cross-sectional study. | April-21 | Turkey | English | Cross-sectional | Ann Clin Microbiol Antimicrob | 7 | 2988 | (99) |
| 100 | Yoshihara et al. | Sars-cov-2 seroprevalence among healthcare workers in general hospitals and clinics in Japan | April-21 | Japan | English | Cross-sectional | Int J Environ Res Public Health | 7 | 2160 | (100) |
| 101 | Faller et al. | Seroprevalence study of SARS-CoV-2 antibodies in healthcare workers following the first wave of the COVID-19 pandemic in a teritary-level hospital in the south of Ireland | May-21 | Ireland | English | Prospective cohort | BMJ | 9 | 503 | (101) |
| 102 | Kataria et al. | Seroprevalence of SARS‑CoV‑2 IgG antibodies and risk factors in health care workers at an academic medical center in Boston, Massachusetts | May-21 | United States | English | Cross-sectional observational | Sci Rep | 10 | 1743 | (102) |
| 103 | Ken-Dror et al. | SARS-CoV-2 antibody seroprevalence in NHS healthcare workers in a large double-sited UK hospital | May-21 | United Kingdom | English | Cross-sectional | J Clin Med | 11 | 3119 | (103) |
| 104 | Scohy et al. | Seroprevalence of SARS-CoV-2 infection in health care workers of a teaching hospital in Belgium: self-reported occupational and household risk factors for seropositivity | May-21 | Belgium | English | Cross-sectional | Diagnostic Microbiology and Infectious Disease | 10 | 3255 | (104) |
| 105 | Hasan et al. | Sero-Prevalence of SARS-CoV-2 antibodies in high-risk populations in Vietnam | June-21 | Vietnam | English | Cross-sectional | Int J Environ Res Public Health | 9 | 148 | (105) |
| 106 | Fukuda et al. | SARS-CoV-2 seroprevalence in healthcare workers at a frontline hospital in Tokyo | June-21 | Japan | English | Cross-sectional observational | Sci Rep | 9 | 4147 | (106) |
| 107 | Mortgat et al. | Prevalence and incidence of anti-SARS-CoV-2 antibodies among healthcare workers in Belgian hospitals before vaccination: a prospective cohort study. | June-21 | Belgium | English | Prospective Cohort | BMJ Open | 10 | 850 | (107) |
| 108 | Shields et al. | COVID-19: Seroprevalence and Vaccine Responses in UK Dental Care Professionals | June-21 | United Kingdom | English | Cross-sectional | J Dent Res | 9 | 1507 | (108) |

# **Supplementary Table 3: Table of outcome data (UAE, 2020-2021)**

| # | Author | Title | Publication Date | Location | Language | Study Design | Journal | Total # HCW | HCW PCR Positive | *HCW Seropositivity* | Hospitalized | ICU Admission | Mortality |
| --- | --- | --- | --- | --- | --- | --- | --- | --- | --- | --- | --- | --- | --- |
| 1 | Fafi-Kremer et al. | Serologic responses to SARS-CoV-2 infection among hospital staff with mild disease in eastern France. | July-20 | France | English | Cross-sectional | EBioMedicine | 160 | 160 (100%) | 159 (99.4%) | - | - | - |
| 2 | Zheng et al. | Characteristics and transmission dynamics of COVID-19 in healthcare workers at a London teaching hospital | July-20 | United Kingdom | English | Cross-sectional | J Hosp Infect | 958 | 498 (60%) | - | - | - | - |
| 3 | Lombardi et al. | Characteristics of 1573 healthcare workers who underwent nasopharyngeal swab testing for SARS-CoV-2 in Milan, Lombardy, Italy | July-20 | Italy | English | Observational Cohort | Clin Microbiol Infect | 1573 | 139 (8.8%) | - | 8 (0.5%) | - | - |
| 4 | Epstude et al. | Seroprevalence of COVID-19 antibodies in the cleaning and oncological staff of a municipal clinic | July-20 | Germany | German/English | Cross-sectional | GMS Hyg Infect Control | 65 | 0 (0%) | 2 (3.1%) | - | - | - |
| 5 | Folgueira et al. | SARS-CoV-2 infection in health care workers in a large public hospital in Madrid, Spain, during March 2020. | July-20 | Spain | English | Retrospective cohort study | J Hosp Infect | 1911 | 213 (11.1%) | - | 11 (0.57%) | 1 (0.05%) | 0 |
| 6 | Sotgiu et al. | SARS-CoV-2 specific serological pattern in healthcare workers of an Italian COVID-19 forefront hospital | July-20 | Italy | English | Cross-sectional | BMC Pulm Med | 202 | 7 (3.5%) | IgM: 29 (14.4%) IgG: 15 (7.4%) | - | - | - |
| 7 | Bampoe et. al. | A Cross-sectional study of immune seroconversion to SARS-CoV-2 in frontline maternity health professionals | August-20 | United Kingdom | English | Cross-sectional | Anaesthesia | 200 | - | 29 (14.5%) | - | - | - |
| 8 | Fill Malfertheiner et al. | Immune response to SARS-CoV-2 in health care workers following a COVID-19 outbreak: A prospective longitudinal study. | August-20 | Germany | English | Prospective longitudinal Cross-sectional | J Clin Virol | 166 | 27 (16.3%) | 48.40% | - | - | - |
| 9 | Fuereder et al. | SARS-CoV-2 seroprevalence in oncology healthcare professionals and patients with cancer at a tertiary care centre during the COVID-19 pandemic. | August-20 | Austria | English | Mixed retrospective/prospective cohort | ESMO Open | 62 | - | 2 (3.2%) | - | - | - |
| 10 | Garcia-Basteiro et al. | Seroprevalence of antibodies against SARS-CoV-2 among health care workers in a large Spanish reference hospital | August-20 | Spain | English | Cross-sectional | Nat Commun | 578 | 15(2.6%) | 54(9.3%) | - | - | - |
| 11 | Harada et al. | Control of a Nosocomial Outbreak of COVID-19 in a University Hospital | August-20 | Japan | English | Observational Cohort | Open Forum Infect Dis | 697 | 52 (7.5%) | - | - | - | - |
| 12 | Hartmann et al. | Coronavirus 2019 (COVID-19) Infections Among Healthcare Workers, Los Angeles County, February - May 2020 | August-20 | United States | English | Cross-sectional | Clin Infect Dis | 5118 | 5118 (100%) | - | 271 (5.3%) | - | 40 (0.78%) |
| 13 | Iversen et al. | Risk of COVID-19 in health-care workers in Denmark: an observational cohort study | August-20 | Denmark | English | Observational Cohort | Lancet Infect Dis | 28,792 | 360 (1.2%) | 1163 (4.04%) | - | - | - |
| 14 | Jeremias et al. | Prevalence of SARS-CoV-2 Infection Among Health Care Workers in a Tertiary Community Hospital | August-20 | United States | English | Cross-sectional | JAMA Intern Med | 3046 | 184 (6.04%) | 167 (5.45%) | - | - | - |
| 15 | Vilela-Estrada et al. | [Clinical manifestations and course of the first six reported cases of COVID-19 in the medical doctors of Peru] | August-20 | Peru | Spanish | Case report | Medwave | *6* | 6 (100%) | - | 0 | 0 | 0 |
| 16 | Lackermair et al. | Infection with SARS-CoV-2 in primary care health care workers assessed by antibody testing | August-20 | Germany | English | Observational Cohort | Fam Pract | 151 | 2 (1.3%) | - | 1 (0.66%) | - | - |
| 17 | Malik et al. | Clinical presentation, management and in-hospital outcome of healthcare personnel with COVID-19 disease | August-20 | Bangladesh | English | Cross-sectional | Cureus | 1409 | 139 (9.8%) | - | 20 (1.4%) | - | 1 |
| 18 | Plebani et al. | SARS-CoV-2 serosurvey in health care workers of the Vento region | August-20 | Italy | English | Cross-sectional | Clin Chem Lab Med | 8,285 | - | 378 (4.6%) | - | - | - |
| 19 | Rizza et al. | High body mass index and night shift work are associated with COVID-19 in health care workers | August-20 | Italy | English | Cross-sectional | J Endocrinol Invest | 1180 | 24 (2%) | 6 (0.5%) | - | - | - |
| 20 | Stock et al. | COVID-19 Infection Among Healthcare Workers: Serological Findings Supporting Routine Testing | August-20 | United States | English | Cross-sectional | Front Med | 98 | 19 (19.4%) | 11 (11.2%) | - | - | - |
| 21 | Dacosta-Urbieta et al. | Seroprevalence of SARS-CoV-2 Among Pediatric Healthcare Workers in Spain | September-20 | Spain | English | Cross-sectional | Frontiers in Pediatr | 175 |  | 7 (4%) | - | - | - |
| 22 | García-Sierra et al. | Descriptive study of the health service workers of a Primary Care Department confined by Covid-19. | September-20 | Spain | Spanish | Cross-sectional | Rev Esp Salud Publica | 1418 | 323 (22.8%) | - | 63 (4.4%) | - | - |
| 23 | Guerrero-Torres et al. | Clinical Characteristics and Mortality of Healthcare Workers with SARS-CoV-2 infection in Mexico City | September-20 | Mexico | English | Cross-sectional | Clin Infect Dis | 16,446 | 16446 (100%) | - | 1310 (8%) | - | 321 (1.9%) |
| 24 | Kassem et al. | SARS-CoV-2 infection among healthcare workers of a gastroenterological service in a tertiary care facility | September-20 | Egypt | English | Cross-sectional | Arab J Gastroenterol | 74 | 10 (13.5%) | 9 (12.2%) | - | - | - |
| 25 | Self et al. | Seroprevalence of SARS-CoV-2 Among Frontline Health Care Personnel in a Multistate Hospital Network - 13 Academic Medical Centers, April-June 2020. | September-20 | United States | English | Cross-sectional | MMWR Morb Mortal Wkly Rep | 3248 | 67 (2.1%) | 194 (6%) | - | - | - |
| 26 | Vandercam et el. | Clinical characteristics and humoral immune response in healthcare workers with COVID-19 in a teaching hospital in Belgium | September-20 | Belgium | English | Retrospective study | J Hosp Infect | 7757 | 183 (data reported on 176) (2.4%) | 109 (1.4%) | 13 (0.2%) | 3 (0.04%) | 1 (0.01%) |
| 27 | Zhang et al. | Factors associated with asymptomatic infection in health-care workers with severe acute respiratory syndrome coronavirus 2 infection in Wuhan, China: a multicentre retrospective cohort study | September-20 | China | English | Retrospective cohort | Clin Microbiol Infect | 8553 | 257 (3%) | 189 (2.2%) | 195 (2.3%) | - | - |
| 28 | Alajmi et al. | COVID-19 infection among healthcare workers in a national healthcare system: The Qatar experience | September-20 | Qatar | English | Cross-sectional | Int J Infect Dis | 16912 | 1799 (10.6%) | - | 278 (1.6%) | 4 (0.02%) | 0 |
| 29 | Misra-Hebert et al. | Impact of the COVID-19 pandemic on healthcare workers' risk of infection and outcomes in a large, integrated health system | September-20 | United States | English | Retrospective cohort study | J Gen Intern Med | 6145 | 551 (9%) | - | 38 (6.9%) | 10 (1.8%) | - |
| 30 | Moscola et al. | Prevalence of SARS-CoV-2 antibodies in health care personnel in the New York City area | September-20 | United States | English | Cohort study | JAMA | 40,329 | 2186 (5.4%) | - | - | - | - |
| 31 | Shah et al. | Risk of hospital admission with coronavirus disease 2019 in healthcare workers and their households: nationwide linkage cohort study | September-20 | United Kingdom | English | Cross-sectional | BMJ | 158,445 | 243 (0.15%) | - | 243 (0.15%) | 30 (0.02%) | 6 (0.004%) |
| 32 | Shields et al. | SARS-CoV-2 seroprevalence and asymptomatic viral carriage in healthcare workers: a Cross-sectional study | September-20 | United Kingdom | English | Cross-sectional | Thorax | 516 | 13 (2.5%) | 126 (24.4%) | - | - | - |
| 33 | Meng et al. | Epidemiological investigation of OHCWs with COVID-19 | September-20 | China | English | Retrospective cohort study | J of Dental Res | 31 | 27 (87.1%) | - | 31 (100%) | - | 1 (3.2%) |
| 34 | Perotti et al. | SARS-CoV-2 antibody prevalence in health care workers of Lodi hospital, the COVID-19 Italian Epicentre | September-20 | Italy | English | Cross-sectional | SSRN Electronic Journal | 2415 | - | 400 (16.5%) | - | - | - |
| 35 | Fell et al. | SARS-CoV-2 Exposure and Infection Among Health Care Personnel - Minnesota, March 6-July 11, 2020. | October-20 | United States | English | Prospective cohort | MMWR Morb Mortal Wkly Rep | 5,374 | 373 (6.9%) | - | - | - | - |
| 36 | Ibrahim et al. | COVID-19 in health-care workers: Testing and outcomes at a Victorian tertiary children's hospital | October-20 | Australia | English | Observational Cohort | J Paediatr Child Health | 1,964 | 11 (0.6%) | - |  | - |  |
| 37 | Kambhampati et al. | COVID-19-Associated Hospitalizations Among Health Care Personnel - COVID-NET, 13 States, March 1-May 31, 2020 | October-20 | United States | English | Cross-sectional | MMWR Morb Mortal Wkly Rep | 438 | 438 (100%) | - | 438 (100%) | 116 (26.5%) | 16 (3.6%) |
| 38 | Rudberg et al. | SARS-CoV-2 exposure, symptoms and seroprevalence in healthcare workers in Sweden | October-20 | Sweden | English | Cross-sectional | Nat Commun | 2149 | - | 410 (19.1%) | - | - | - |
| 39 | Akyala et al. | Severe Acute Respiratory Syndrome Coronavirus 2 (SARS-CoV-2) infection among health care workers in Nasarawa State, Nigeria: implications for infection prevention and control measures | October-20 | Nigeria | English | Case series analysis | Pan Afr Med J | 648 | 134 (20.7%) | - |  | - | 7 (1.1%) |
| 40 | Augusto et al. | Healthcare Workers Bioresource: Study outline and baseline characteristics of a prospective healthcare worker cohort to study immune protection and pathogenesis in COVID-19 | October-20 | United Kingdom | English | Prospective cohort | Wellcome Open Res | 400 | 28 (7%) | - | - | - | - |
| 41 | Barrett et al. | Prevalence of SARS-CoV-2 infection in previously undiagnosed health care workers in New Jersey, at the onset of the U.S. COVID-19 pandemic | November-20 | United States | English | Prospective cohort | BMC Infect Dis | 546 | 40 out of 546 (7.3%) | - | - | - | - |
| 42 | Brant-Zawadzski et al. | SARS-CoV-2 antibody prevalence in health care workers: Preliminary report of a single center study | November-20 | United States | English | Cross-sectional | PLoS ONE | 2924 | - | 31 (1.1%) | - | - | - |
| 43 | Goenka et al. | Seroprevalence of COVID-19 Amongst Health Care Workers in a Tertiary Care Hospital of a Metropolitan City from India. | November-20 | India | English | Cross-sectional | J Assoc Physicians India | 1122 | - | 134 (11.9%) | - | - | - |
| 44 | Gras-Valenti et al. | Sero-epidemiological study of SARS-CoV-2 infection among healthcare personnel in a healthcare department | November-20 | Spain | Spanish | Cross-sectional | Enferm infecc Microbiol Clin | 4179 | - | 274 (6.6%) | - | - | - |
| 45 | Ko et al. | Serologic Evaluation of Healthcare Workers Caring for COVID-19 Patients in the Republic of Korea | November-20 | South Korea | English | Cross-sectional | Front Microbiol | 432 | 0 | 1 (0.2%) | - | - | - |
| 46 | Eren et al. | Evaluation of health care workers with COVID-19. | November-20 | Turkey | Turkish | Retrospective Cross-sectional | Klimik Derg. | 1325 | 64 (4.8%) | - | - | - | - |
| 47 | Mahajan et al. | Prevalence and clinical presentation of COVID-19 among healthcare workers at a dedicated hospital in India | November-20 | India | English | Observational retrospective monocentric study | J Assoc Physicians India | 3711 | 413 (11%) | - | - | 35 (0.9%) | 400% |
| 48 | Martin et al. | Demographic and occupational determinants of anti-SARS-CoV-2 IgG seropositivity in hospital staff | November-20 | United Kingdom | English | Case-series | J Public Health (Oxf) | 10662 | 205 (25%) | 1148 (10.8%) | - | - | - |
| 49 | Rubbi et al. | Healthcare personnel exposure to COVID - 19: an observational study on quarantined positive workers | November-20 | Italy | English | Retrospective observational study | Acta Biomedica | 3565 | 93 (2.6%) | - | - | - | - |
| 50 | Abohamr et al. | Clinical characteristics and in-hospital outcome of medical staff infected with COVID-19 in Saudi Arabia | December-20 | Saudi Arabia | English | Retrospective Cross-sectional | Saudi Med J. | 108 | 108 (100%) | - | 68 (63%) | 18 (26.5%) | 5 (4.6%) |
| 51 | Barallat et al. | Seroprevalence of SARS-CoV-2 IgG specific antibodies among healthcare workers in the Northern Metropolitan Area of Barcelona, Spain, after the first pandemic wave | December-20 | Spain | English | Prospective Cross-sectional | PLoS ONE | 7563 | 341 (43.8%) | - | 59 (0.8%) | - | - |
| 52 | Buonafire et al. | High prevalence of SARS-CoV-2 infection among symptomatic healthcare workers in a large university tertiary hospital in São Paulo, Brazil | December-20 | Brazil | English | Cross-sectional | BMC Infect Dis | 295 | 125 (42.4%) | - | 9 (3%) | - | 2 (0.7%) |
| 53 | Foo et al. | COVID-19 testing strategy in response to infection among healthcare workers in a large non-COVID-designated hospital. | December-20 | Malaysia | English | Cross-sectional | Hosp Pract | 3336 | 22 (0.6%) | - | - | - | - |
| 54 | Fujita et al. | Quantitative SARS-CoV-2 Antibody Screening of Healthcare Workers in the Southern Part of Kyoto City During the COVID-19 Pre-pandemic Period | December-20 | Japan | English | Cross-sectional | Front Public Health | 92 | - | IgG: positive 5 (5.4%) | - | - | - |
| 55 | Galán et al. | Hospital-Wide SARS-CoV-2 seroprevalence in health care workers in a Spanish teaching hospital | December-20 | Spain | English | Cross-sectional | Enferm Infecc Microbiol Clin (Engl Ed) | 2590 | 306 (11.8%) | 818 (31.6%) | 25 (1%) | - | - |
| 56 | Insúa et al. | Seroprevalence of SARS-CoV-2 antibodies among physicians from a children's hospital | December-20 | Argentina | English | Cross-sectional | Arch Argent Pediatr | 116 | - | - | - | - | - |
| 57 | Kim et al. | Comparison of COVID-19 infections among healthcare workers and non-healthcare workers | December-20 | United States | English | Cross-sectional | PLoS One | 193 | 193 positive HCW 100% | - | 38 (19.7%) | 7 (3.6%) | 2 (1%) |
| 58 | Krastinova et al. | Household transmission and incidence of positive SARS-CoV-2 RT-PCR in symptomatic healthcare workers, clinical course and outcome: a French hospital experience | December-20 | France | English | Cross-sectional | Occup Environ Med | 314 | 110 (35%) | - | 9 (2.9%) | 2 (0.6%) | 0 |
| 59 | Schwarts et al. | Epidemiology, clinical characteristics, household transmission, and lethality of severe acute respiratory syndrome coronavirus-2 infection among healthcare workers in Ontario, Canada | December-20 | Canada | English | Cross-sectional | PLOS one | 552,560 | 7050 (1.3%) | - | 179 (0.03%) | 56 (0.01%) | 12 (0.002%) |
| 60 | Kantele et al. | SARS-CoV-2 infections among healthcare workers at Helsinki University Hospital, Finland, spring 2020: Serosurvey, symptoms and risk factors | December-20 | Finland | English | Cross-sectional | Travel Med Infect Dis | 1095 | 36 (3.3%) | 33 (3%) | 1 (0.09%) | 1 (0.09%) | - |
| 61 | Alshahrani et al. | Prevalence of the SARS-CoV-2 infection among post-quarantine healthcare workers | December-20 | Saudi Arabia | English | Prospective Cross-sectional | J Multidiscip Healthc | 301 | 18 (6%) | - | - | - | - |
| 62 | Coppeta et al. | Contact Screening for Healthcare Workers Exposed to Patients with COVID-19 | December-20 | Italy | English | Retrospective Cross-sectional | Int J Environ Res Public Health | 1006 | 12 (1.19%) | - | - | - | - |
| 63 | Li et al. | Mild manifestations of COVID-19 in healthcare workers | December-20 | China | English | Retrospective case series study | PLOS Negl Trop Dis | 56 | 56 (100%) | - | 56 (100%) | - | 1 (2%) |
| 64 | Mandić-Rajčević et al. | Source and symptoms of COVID-19 among hospital workers in Milan | December-20 | Italy | English | Cross-sectional | Occup Med | 185 | 185 (100%) | - | - | - | - |
| 65 | Lawati et al. | Risk of COVID-19 Infection in Healthcare Workers Exposed During Use of Non-invasive Ventilation in a Tertiary Care Hospital in Oman | December-20 | Oman | English | Prospective Cohort | Oman Med J | 38 | 34 (89.5%) |  | 0 | - | - |
| 66 | Escribano Ceruelo et al. | Seroprevalence of antibodies against SARS-CoV-2 among health care workers in a pediatric monographic hospital in Madrid (Spain) | January-21 | Spain | Spanish | Prospective cohort | Enferm infecc Microbiol Clin | 1292 |  | 222 (17.2%) | - | - | - |
| 67 | Goldblatt et al. | Cross-sectional prevalence of SARS-CoV-2 antibodies in healthcare workers in paediatric facilities in eight countries | January-21 | Europe & South Africa | English | Cross-sectional | J Hosp Infect | 4114 | 50 (1.2%) Austria 0 Estonia 0 Greece 0 Latvia 0 Lithuania 2 (0.05%) Romania 1 (0.02%) South Africa 17 (0.4%) UK 30 (0.7%) | 488 (11.9%) Austria 0 Estonia 0 Greece 1 (0.02%) Latvia 0 Lithuania 2 (0.05%) Romania 1 (0.02%) South Africa 23 (0.6%) UK 461 (11.2%) | - | - | - |
| 68 | Gupta et al. | Seroprevalence of antiodies to SARS-CoV-2 in healthcare workers and implications of infection control practice in India | January-21 | India | English | Cross-sectional | Indian J Med Res | 3739 | 193 (Out of 968 who underwent testing) (5.2%) | 487 (13%) | - | 0 | - |
| 69 | Huete-Pérez et al. | First report on prevalence of SARS-CoV-2 infection among health-care workers in Nicaragua | January-21 | Nicaragua | English | Cross-sectional | PLOS ONE | 402 | 122 (30.3%) | - | - | - | - |
| 70 | Johnson et al. | Prevalence of SARS-CoV-2 antibodies among North Dakota community pharmacy personnel: A seroprevalence survey | January-21 | United States | English | Cross-sectional | J Am Pharm Assoc | 247 |  | 36 (15.6%) | - | - | - |
| 71 | Abdelmoniem et al | SARS-Cov-2 infection among asymptomatic healthcare workers of the emergency department in a tertiary care facility | January-21 | Egypt | English | Cross-sectional | J Clin Virol | 203 | 29 (14.3%) | 37 (18.2%) | - | - |  |
| 72 | Abdi et al | Preliminary findings of COVID-19 infection in health workers in Somalia: A reason for concern | January-21 | Somalia | English | Cross-sectional | Int J Infect Dis | 311 | 191 (61.4%) | - | - | - | 2 (0.6%) |
| 73 | Nicolas et al. | A Prospective Cohort of SARS-CoV-2-Infected Health Care Workers: Clinical Characteristics, Outcomes, and Follow-up Strategy | Januray-21 | Spain | English | Prospective Cohort | Open Forum Infect Dis | 590 | 590 (100%) | - | 6 (1.02%) | 0 | 0 |
| 74 | Olayanju et al. | SARS-CoV-2 seropositivity in asymptomatic frontline health workers in Ibadan, Nigeria | Januray-21 | Nigeria | English | Cross-sectional | Am J Trop Med Hyg | 133 | - | 60 (45.1%) | - | - | - |
| 75 | Olmos et al. | SARS-CoV-2 infection in asymptomatic healthcare workers at a clinic in Chile. | Janurary 21 | Chile | English | Cross-sectional | PLOS One | 413 | 14 (3.4%) | - | - | - | - |
| 76 | Platten et al. | Prevalence of SARS-CoV-2 in employees of a general hospital in Northrhine-Westphalia, Germany | Janurary 21 | Germany | German | Cross-sectional | Dtsch Med Wochenschr | 1363 | 19 (1.4%) | - | - | - | - |
| 77 | Díez-Manglano et al. | Risk of death of healthcare workers hospitalized due to COVID-19 | February-21 | Spain | English | observational cohort | PLOS ONE | 419 | 419 (100%) | - | 419 (100%) | 44 (10.5%) | 3 (0.7%) |
| 78 | Comelli et al. | Serological Response to SARS-CoV-2 in Health Care Workers Employed in a Large Tertiary Hospital in Lombardy, Northern Italy | February-21 | Italy | English | Retrospective Observatioal | Microorganisms | 1893 | - | 433 (22.9%) | 20 (1.06%) | 4 (0.2%) | - |
| 79 | Jungo et al. | Prevalence and risk indicators of first-wave COVID-19 among oral health-care workers: A French epidemiological survey | Febuary-21 | France | English | Cross-sectional | PLOS ONE | 4172 | 79 (Out of 199 tested) 1.9%) |  | - | - | - |
| 80 | Khan et al. | SARS-CoV-2 Seroprevalence Among Healthcare Workers by Workplace Exposure Risk in Kashmir, India | Febuary-21 | India | English | Cross-sectional observational | J Hosp Med | 2915 | 29 (1%) | 73 (2.5%) | - | - | - |
| 81 | Lankarani et al. | Prevalence of Anti-SARS-CoV-2 Antibody in Hospital Staff in Double-Center Setting: A Preliminary Report of a Cohort Study From Iran | Febuary-21 | Iran | English | Cohort | Shiraz E-Med J | 494 | - | 29 (5.9%) | - | - | - |
| 82 | Ledda et al. | SARS-CoV-2 Seroprevalence Post-First Wave among Primary Care Physicians in Catania (Italy) | Febuary-21 | Italy | English | Cross-sectional | Trop Med Infect Dis | 133 | - | 4 (3%) | - | - | - |
| 83 | Lumley et al. | Antibody status and incidence of SARS-CoV-2 infection in health care workers. | Febuary-21 | United Kingdom | English | Prospective Cohort | NEJM | 1177 | 239 (Out of 358 that got tested) (20.3%) | 1177 (100%) | - | - | - |
| 84 | Handal et al. | Comparison of SARS-CoV-2 infections in healthcare workers with high and low exposures to COVID-19 patients in a Norwegian University Hospital | Feburary-21 | Norway | English | Cross-sectional | J Infect Dis | 360 | - | 12 (3.3%) | - | - | - |
| 85 | Jacob et al. | Risk factors associated with SARS-CoV-2 seropositivity among US health care personnel | March-21 | United States | English | Cross-sectional | JAMA | 24749 | - | 1080 (4.4%) | - | - | - |
| 86 | Nygren et al. | Association between SARS-CoV-2 and exposure risks in health care workers and university employees–a Cross-sectional study | Mar-21 | Sweden | English | Cross-sectional | J Infect Dis | 271 | 14 (5.2%) | 16 (5.9%) | - | - | - |
| 87 | Airoldi et al | High Seroprevalence of SARS-CoV-2 among Healthcare Workers in a North Italy Hospital | March-21 | Italy | English | Cross-sectional | Int J Environ Res Public Health | 2250 | - | 385 (17.1%) | - | - | - |
| 88 | Alkurt et al | Seroprevalence of coronavirus disease 2019 (COVID-19) among health care workers from three pandemic hospitals in Turkey | March-21 | Turkey | English | Cross-sectional | PLOS ONE | 932 | 119 (12.8%) | 115 (12.3%) | - | - | - |
| 89 | Johnson et al. | SARS-CoV-2 RT-PCR positivity and antibody prevalence among asymptomatic hospital-based health care workers | March-21 | United States | English | Cross-sectional | J Clin Virol | 374 | 1 (0.3%) | 9 (2.4%) | - | - | - |
| 90 | Mostafa et al. | SARS-CoV-2 seroconversion among 4040 Egyptian healthcare workers in 12 resource-limited healthcare facilities: A prospective cohort study. | March-21 | Egypt | English | Prospective cohort | Int J Infect Dis | 2282 | - | 100 (4.4%) | - | - | - |
| 91 | Oliveira et al. | SARS-Cov-2 seroprevalence and risk factors among health care workers: Estimating the risk of COVID-19 dedicated units | March-21 | Brazil | English | Prospective Cross-sectional | Am J Infect Control | 1996 | - | 110 (5.5%) | - | - | - |
| 92 | Özdemir at al. | SARS-CoV-2 seroprevalence among healthcare workers: Retrospective analysis of the data from a university hospital in Turkey | March-21 | Turkey | Turkish | Retrospective cohort | Mikrobiyol Bul | 774 | - | 57 (7.4%) | - | - | - |
| 93 | Purswani et al. | SARS-CoV-2 Seroprevalence Among Healthcare Workers by Job Function and Work Location in a New York Inner-City Hospital | March-21 | United States | English | Cross-sectional | J Hosp Med | 2749 | - | 831 (30.2%) | - | - | - |
| 94 | Sabetian et al. | COVID-19 infection among healthcare workers: a Cross-sectional study in southwest Iran | March-21 | Iran | English | Cross-sectional | Virol J | 273 | 273 (100%) | - | - | 0 | 0 |
| 95 | Abbas et al | Seroprevalence of SARS-CoV-2 Antibodies Among Health Care Personnel at a Health Care System in Pakistan | April-21 | Pakistan | English | Prospective Cohort | Asia Pac J | 2162 | - | 857 (39.6%) | - | - | - |
| 96 | Colaneri et al. | The experience of the health care workers of a severely hit SARS-CoV-2 referral Hospital in Italy; incidence, clinical course and modifiable risk factor for COVID-19 infection | April-21 | Italy | English | Cross-sectional | J Public Health | 1447 | 164 (11.3%) |  | 9 (0.6%) | - | 0 |
| 97 | Kindgen-Milles et al. | Prevalence of SARS-COV-2 positivity in 516 German intensive care and emergency physicians studied by seroprevalence of antibodies National Covid Survey Germany (NAT-COV-SURV) | April-21 | Germany | English | Cross-sectional | PLOS ONE | 516 | - | 16 (3.1%) | - | - | - |
| 98 | Morris et al. | Prevalence of SARS-CoV-2 antibodies in pediatric healthcare workers | April-21 | United States | English | Prospective Longitudinal Cohort | Int J Infect Dis | 642 | 6 (0.9%) | 26 (4%) | 0 | - | - |
| 99 | Ogutlu et al. | Novel coronavirus seropositivity and related factors among healthcare workers at a university hospital during the prevaccination period: a Cross-sectional study. | April-21 | Turkey | English | Cross-sectional | Ann Clin Microbiol Antimicrob | 2988 | - | 108 (3.6%) | - | - | - |
| 100 | Yoshihara et al. | Sars-cov-2 seroprevalence among healthcare workers in general hospitals and clinics in Japan | April-21 | Japan | English | Cross-sectional | Int J Environ Res Public Health | 2160 | - | 36 (1.7%) | - | - | - |
| 101 | Faller et al. | Seroprevalence study of SARS-CoV-2 antibodies in healthcare workers following the first wave of the COVID-19 pandemic in a teritary-level hospital in the south of Ireland | May-21 | Ireland | English | Prospective cohort | BMJ | 503 | 99 (19.7%) | 78 (15.5%) | 1 (0.2%) | - | - |
| 102 | Kataria et al. | Seroprevalence of SARS‑CoV‑2 IgG antibodies and risk factors in health care workers at an academic medical center in Boston, Massachusetts | May-21 | United States | English | Cross-sectional observational | Sci Rep | 1743 | 85 (4.9%) | 95 (5.4%) | - | - | - |
| 103 | Ken-Dror et al. | SARS-CoV-2 antibody seroprevalence in NHS healthcare workers in a large double-sited UK hospital | May-21 | United Kingdom | English | Cross-sectional | J Clin Med | 3119 |  | 613 (19.6%) | - | - | - |
| 104 | Scohy et al. | Seroprevalence of SARS-CoV-2 infection in health care workers of a teaching hospital in Belgium: self-reported occupational and household risk factors for seropositivity | May-21 | Belgium | English | Cross-sectional | Diagnostic Microbiology and Infectious Disease | 3255 | 515 (15.8%) | 253 (7.8%) | - | - | - |
| 105 | Hasan et al. | Sero-Prevalence of SARS-CoV-2 antibodies in high-risk populations in Vietnam | June-21 | Vietnam | English | Cross-sectional | Int J Environ Res Public Health | 148 | - | 0 | - | - | - |
| 106 | Fukuda et al. | SARS-CoV-2 seroprevalence in healthcare workers at a frontline hospital in Tokyo | June-21 | Japan | English | Cross-sectional observational | Sci Rep | 4147 | - | 14 (0.3%) | - | - | - |
| 107 | Mortgat et al. | Prevalence and incidence of anti-SARS-CoV-2 antibodies among healthcare workers in Belgian hospitals before vaccination: a prospective cohort study. | June-21 | Belgium | English | Prospective Cohort | BMJ Open | 850 | 72 (8.5%) |  | - | - | - |
| 108 | Shields et al. | COVID-19: Seroprevalence and Vaccine Responses in UK Dental Care Professionals | June-21 | United Kingdom | English | Cross-sectional | J Dent Res | 1507 | - | 246 (16.3%) | - | - | - |

# **Supplementary Figure 1. Forest Plots of Outcomes amongst Total Healthcare Workers Period 1: July-December 2020**

# **A. Co-morbidities (UAE, 2020-2021)**


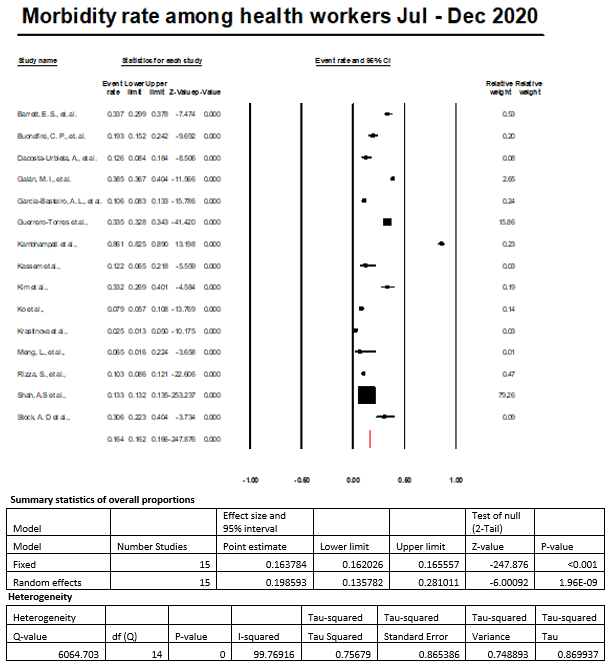


# **B. PCR Positivity Forest Plot and Funnel Plot (UAE, 2020-2021)**


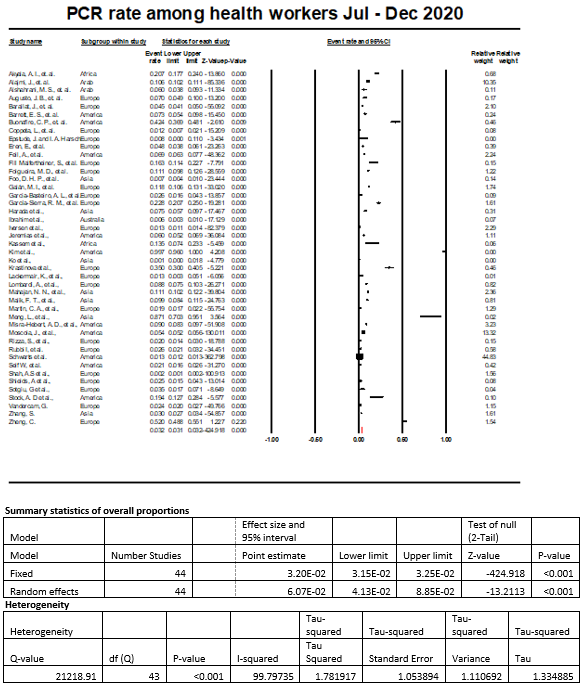

# **C. Seropositivity (UAE, 2020-2021)**


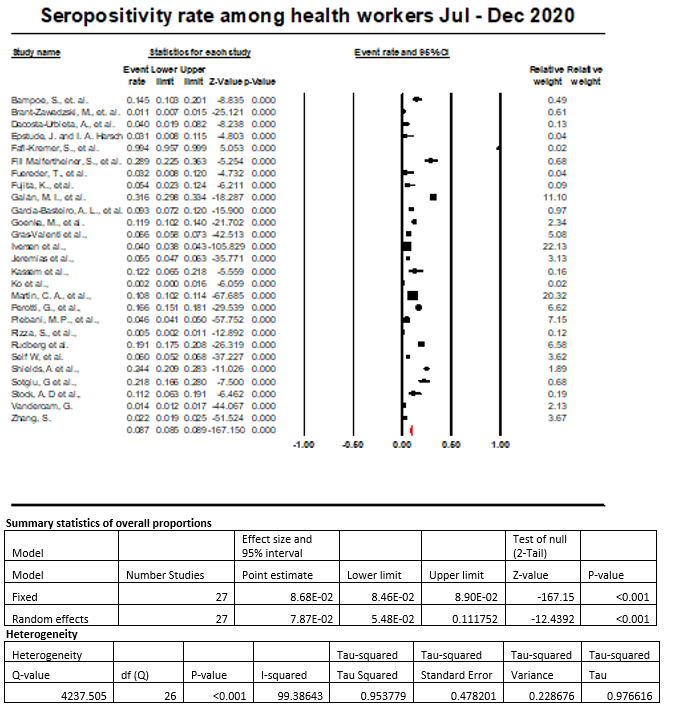


# **D. Hospitalizations (UAE, 2020-2021)**


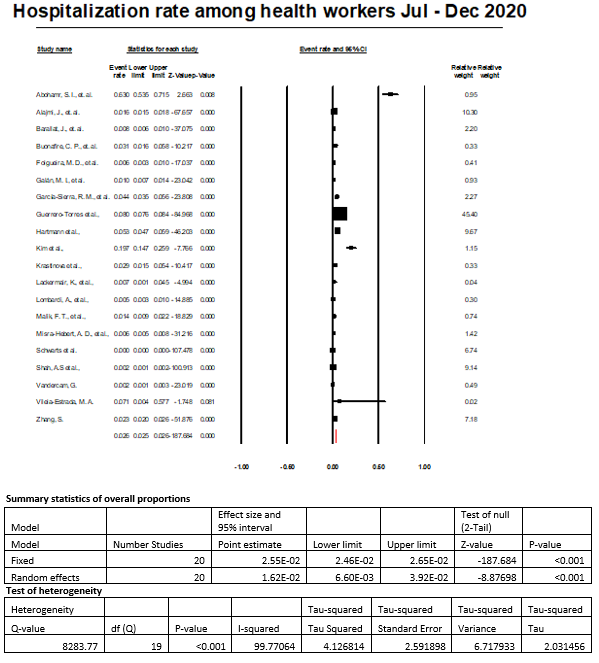


# **E. ICU Admission (UAE, 2020-2021)**


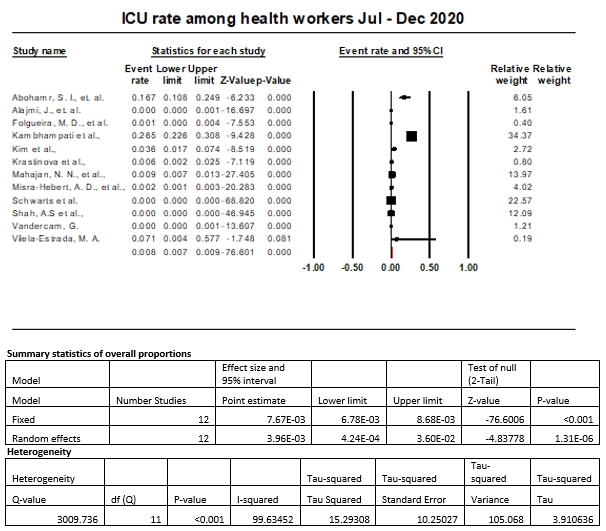


# **F. Mortality (UAE, 2020-2021)**


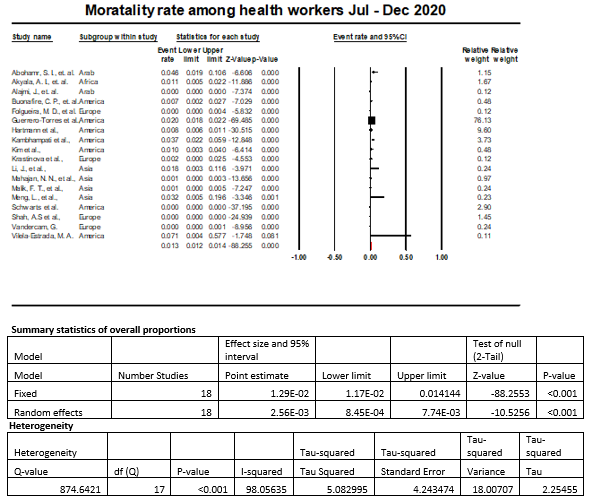


# **Supplementary Figure 2. Forest Plots of Outcomes Amongst Total Healthcare Workers Time Period 2: January-June 2021**

# **A. Co-morbidities (UAE, 2020-2021)**


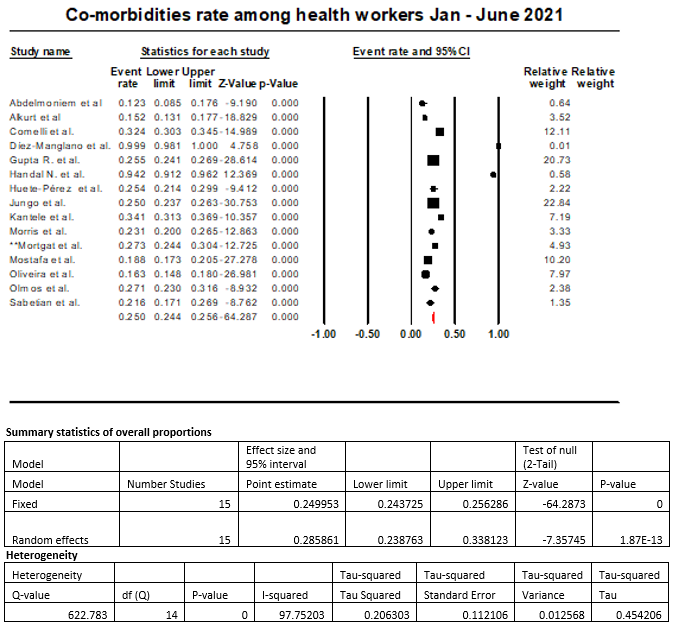


# **B. PCR positivity (UAE, 2020-2021)**


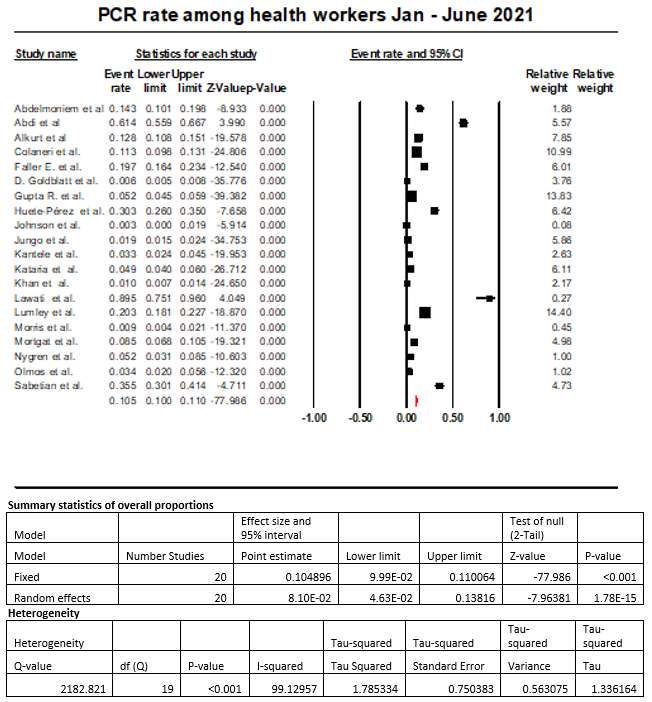


# C**. Seropositivity (UAE, 2020-2021)**


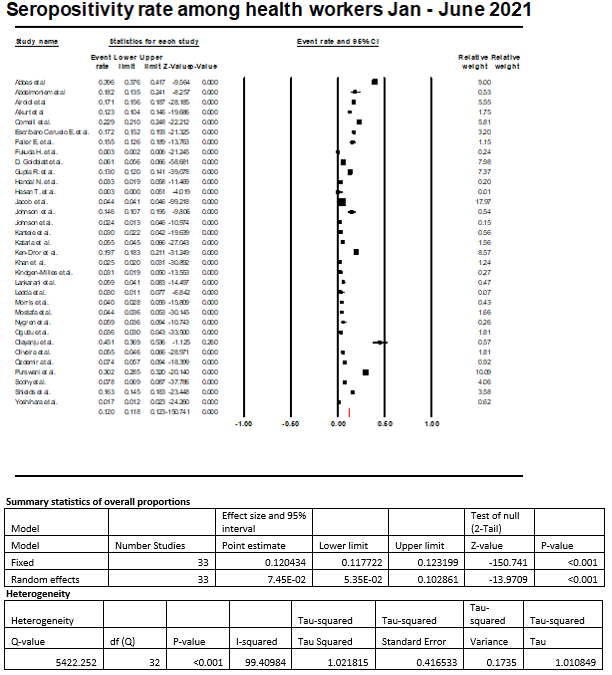


# **D. Hospitalizations (UAE, 2020-2021)**


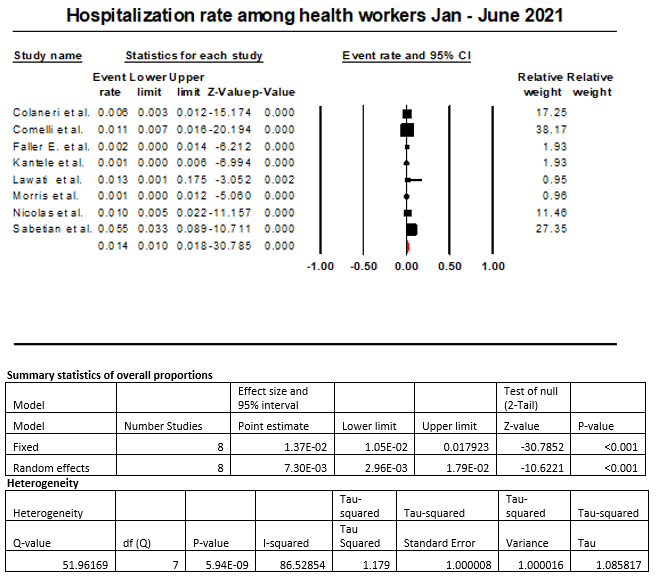


# **E. ICU admissions (UAE, 2020-2021)**


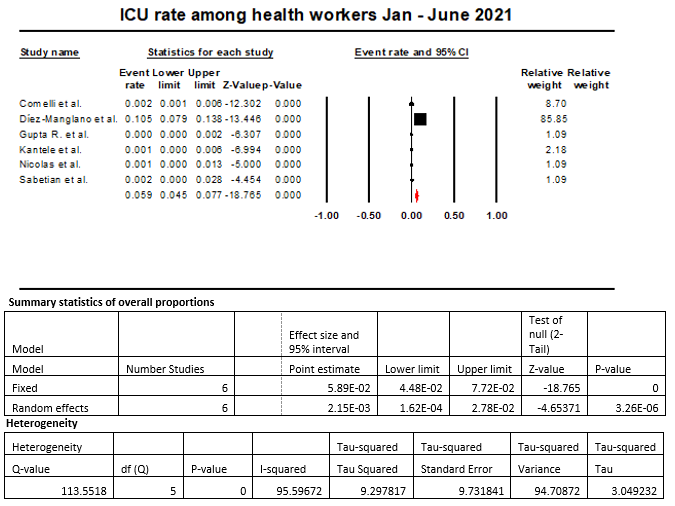


**F. Mortality (UAE, 2020-2021)**


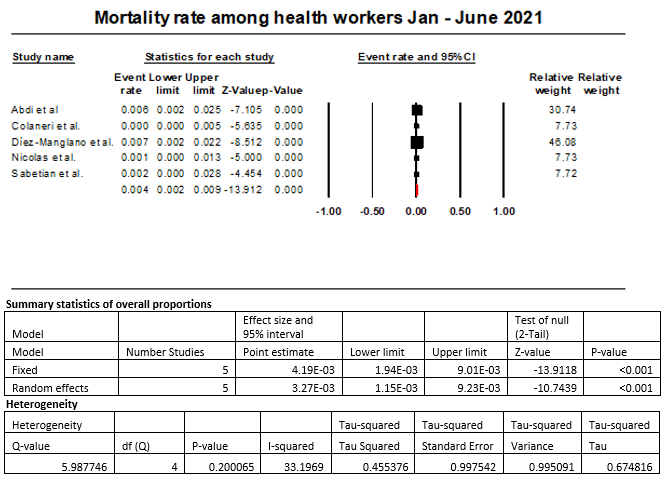


# **G. Vaccinations (UAE, 2020-2021)**


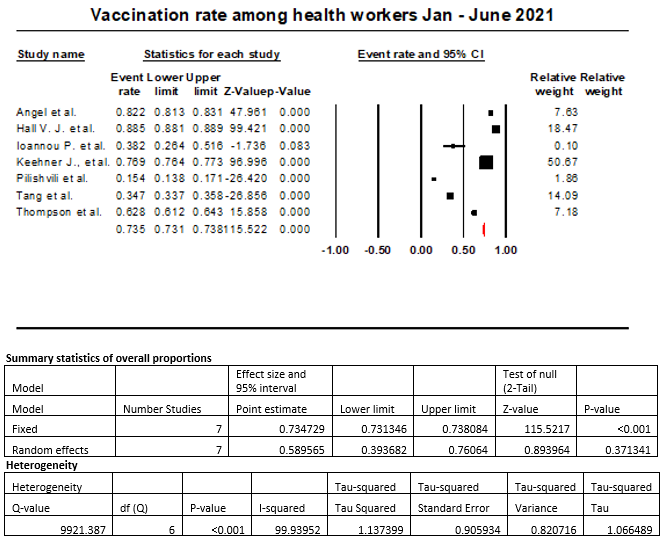


# **H. Global comparison – PCR positivity (UAE, 2020-2021)**


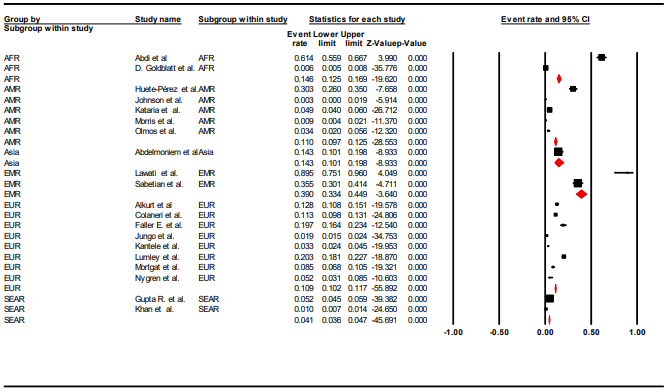


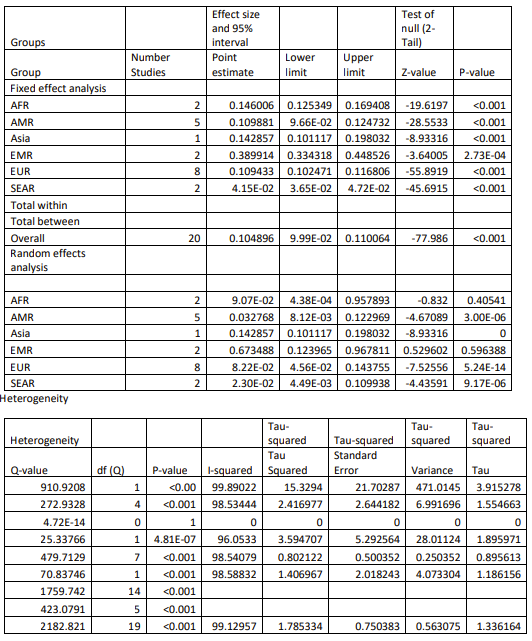


AFR: African Region

AMR: American Region

EMR: Eastern Mediterranean Region

EUR: Europe Region

SEAR: South East Asian Region

# **References**

1. Fafi-Kremer S, Bruel T, Madec Y, Grant R, Tondeur L, Grzelak L, et al. Serologic responses to SARS-CoV-2 infection among hospital staff with mild disease in eastern France. EBioMedicine. 2020;59.

2. Zheng C, Hafezi-Bakhtiari N, Cooper V, Davidson H, Habibi M, Riley P, et al. Characteristics and transmission dynamics of COVID-19 in healthcare workers at a London teaching hospital. J Hosp Infect. 2020;106(2):325-9.

3. Lombardi A, Consonni D, Carugno M, Bozzi G, Mangioni D, Muscatello A, et al. Characteristics of 1573 healthcare workers who underwent nasopharyngeal swab testing for SARS-CoV-2 in Milan, Lombardy, Italy. Clin Microbiol Infect. 2020;26(10):1413.e9-.e13.

4. Epstude J, Harsch IA. Seroprevalence of COVID-19 antibodies in the cleaning and oncological staff of a municipal clinic. GMS Hyg Infect Control. 2020;15:Doc18.

5. Folgueira MD, Munoz-Ruiperez C, Alonso-Lopez MA, Delgado R. SARS-CoV-2 infection in health care workers in a large public hospital in Madrid, Spain, during March 2020. medRxiv. 2020.

6. Sotgiu G, Barassi A, Miozzo M, Saderi L, Piana A, Orfeo N, et al. SARS-CoV-2 specific serological pattern in healthcare workers of an Italian COVID-19 forefront hospital. BMC Pulm Med. 2020;20(1):203.

7. Bampoe S, Lucas DN, Neall G, Sceales P, Aggarwal R, Caulfield K, et al. A cross-sectional study of immune seroconversion to SARS-CoV-2 in frontline maternity health professionals. Anaesthesia. 2020;75(12):1614-9.

8. Fill Malfertheiner S, Brandstetter S, Roth S, Harner S, Buntrock-Döpke H, Toncheva AA, et al. Immune response to SARS-CoV-2 in health care workers following a COVID-19 outbreak: A prospective longitudinal study. J Clin Virol. 2020;130:104575.

9. Fuereder T, Berghoff AS, Heller G, Haslacher H, Perkmann T, Strassl R, et al. SARS-CoV-2 seroprevalence in oncology healthcare professionals and patients with cancer at a tertiary care centre during the COVID-19 pandemic. ESMO Open. 2020;5(5):e000889.

10. Garcia-Basteiro AL, Moncunill G, Tortajada M, Vidal M, Guinovart C, Jiménez A, et al. Seroprevalence of antibodies against SARS-CoV-2 among health care workers in a large Spanish reference hospital. Nat Commun. 2020;11(1):3500.

11. Harada S, Uno S, Ando T, Iida M, Takano Y, Ishibashi Y, et al. Control of a Nosocomial Outbreak of COVID-19 in a University Hospital. Open Forum Infect Dis. 2020;7(12):1-9.

12. Hartmann S, Rubin Z, Sato H, K OY, Terashita D, Balter S. Coronavirus 2019 (COVID-19) Infections Among Healthcare Workers, Los Angeles County, February - May 2020. Clin Infect Dis. 2020.

13. Iversen K, Bundgaard H, Hasselbalch RB, Kristensen JH, Nielsen PB, Pries-Heje M, et al. Risk of COVID-19 in health-care workers in Denmark: an observational cohort study. Lancet Infect Dis. 2020;20(12):1401-8.

14. Jeremias A, Nguyen J, Levine J, Pollack S, Engellenner W, Thakore A, et al. Prevalence of SARS-CoV-2 Infection Among Health Care Workers in a Tertiary Community Hospital. JAMA Intern Med. 2020.

15. Vilela-Estrada MA, Benites-Flores IR, García-Saavedra MB, Mejia CR. [Clinical manifestations and course of the first six reported cases of COVID-19 in the medical doctors of Peru]. Medwave. 2020;20(7):e7994.

16. Lackermair K, William F, Grzanna N, Lehmann E, Fichtner S, Kucher HB, et al. Infection with SARS-CoV-2 in primary care health care workers assessed by antibody testing. Fam Pract. 2020.

17. Malik FT, Ishraquzzaman M, Kalimuddin M, Choudhury S, Ahmed N, Badiuzzaman M, et al. Clinical Presentation, Management and In-Hospital Outcome of Healthcare Personnel With COVID-19 Disease. Cureus. 2020;12(8):e10004.

18. Plebani MP, Padoan A, Fedeli U, Schievano E, Vecchiato E, Lippi G, et al. SARS-CoV-2 serosurvey in health care workers of the Veneto Region. Clin Chem Lab Med. 2020;58(12):2107-11.

19. Rizza S, Coppeta L, Grelli S, Ferrazza G, Chiocchi M, Vanni G, et al. High body mass index and night shift work are associated with COVID-19 in health care workers. J Endocrinol Invest. 2020:1-5.

20. Stock AD, Bader ER, Cezayirli P, Inocencio J, Chalmers SA, Yassari R, et al. COVID-19 Infection Among Healthcare Workers: Serological Findings Supporting Routine Testing. Front Med. 2020;7.

21. Dacosta-Urbieta A, Rivero-Calle I, Pardo-Seco J, Redondo-Collazo L, Salas A, Gomez-Rial J, et al. Seroprevalence of SARS-CoV-2 Among Pediatric Healthcare Workers in Spain. Front Pediatr. 2020;8:547.

22. García-Sierra RM, Badia Perich E, Manresa Dominguez JM, Moreno Millan N, Sabaté Cintas V, Romero Martínez M, et al. [Descriptive study of the health service workers of a Primary Care Department confined by Covid-19.]. Rev Esp Salud Publica. 2020;94.

23. Guerrero-Torres L, Caro-Vega Y, Crabtree-Ramírez B, Sierra-Madero JG. Clinical Characteristics and Mortality of Healthcare Workers with SARS-CoV-2 infection in Mexico City. Clin Infect Dis. 2020.

24. Kassem AM, Talaat H, Shawky S, Fouad R, Amer K, Elnagdy T, et al. SARS-CoV-2 infection among healthcare workers of a gastroenterological service in a tertiary care facility. Arab J Gastroenterol. 2020;21(3):151-5.

25. Self WH, Tenforde MW, Stubblefield WB, Feldstein LR, Steingrub JS, Shapiro NI, et al. Seroprevalence of SARS-CoV-2 Among Frontline Health Care Personnel in a Multistate Hospital Network - 13 Academic Medical Centers, April-June 2020. MMWR Morb Mortal Wkly Rep. 2020;69(35):1221-6.

26. Vandercam G, Simon A, Scohy A, Belkhir L, Kabamba B, Rodriguez-Villalobos H, et al. Clinical characteristics and humoral immune response in healthcare workers with COVID-19 in a teaching hospital in Belgium. J Hosp Infect. 2020;106(4):713-20.

27. Zhang S, Guo M, Wu F, Xiong N, Ma Y, Wang Z, et al. Factors associated with asymptomatic infection in health-care workers with severe acute respiratory syndrome coronavirus 2 infection in Wuhan, China: a multicentre retrospective cohort study. Clin Microbiol Infect. 2020;26(12):1670-5.

28. Alajmi J, Jeremijenko AM, Abraham JC, Alishaq M, Concepcion EG, Butt AA, et al. COVID-19 infection among healthcare workers in a national healthcare system: The Qatar experience. Int J Infect Dis. 2020;100:386-9.

29. Misra-Hebert AD, Jehi L, Ji X, Nowacki AS, Gordon S, Terpeluk P, et al. Impact of the COVID-19 Pandemic on Healthcare Workers' Risk of Infection and Outcomes in a Large, Integrated Health System. J Gen Intern Med. 2020;35(11):3293-301.

30. Moscola J, Sembajwe G, Jarrett M, Farber B, Chang T, McGinn T, et al. Prevalence of SARS-CoV-2 Antibodies in Health Care Personnel in the New York City Area. Jama. 2020;324(9):893-5.

31. Shah ASV, Wood R, Gribben C, Caldwell D, Bishop J, Weir A, et al. Risk of hospital admission with coronavirus disease 2019 in healthcare workers and their households: nationwide linkage cohort study. BMJ. 2020;371:m3582.

32. Shields A, Faustini SE, Perez-Toledo M, Jossi S, Aldera E, Allen JD, et al. SARS-CoV-2 seroprevalence and asymptomatic viral carriage in healthcare workers: a cross-sectional study. Thorax. 2020;75(12):1089-94.

33. Meng L, Ma B, Cheng Y, Bian Z. Epidemiological Investigation of OHCWs with COVID-19. J Dent Res. 2020;99(13):1444-52.

34. Perotti G, Baracco A, Filippin A, Anesi A, Beccarini V, Raimondi L, et al. SARS-CoV-2 Antibody Prevalence in Health Care Workers of Lodi Hospital, the COVID-19 Italian Epicentre. Available at SSRN 3650227. 2020.

35. Fell A, Beaudoin A, D'Heilly P, Mumm E, Cole C, Tourdot L, et al. SARS-CoV-2 Exposure and Infection Among Health Care Personnel - Minnesota, March 6-July 11, 2020. MMWR Morb Mortal Wkly Rep. 2020;69(43):1605-10.

36. Ibrahim LF, Cheng DR, Babl FE, Bryant PA, Crawford NW, Daley AJ, et al. COVID-19 in health-care workers: Testing and outcomes at a Victorian tertiary children's hospital. J Paediatr Child Health. 2020;56(10):1642-4.

37. Kambhampati AK, O'Halloran AC, Whitaker M, Magill SS, Chea N, Chai SJ, et al. COVID-19-Associated Hospitalizations Among Health Care Personnel - COVID-NET, 13 States, March 1-May 31, 2020. MMWR Morb Mortal Wkly Rep. 2020;69(43):1576-83.

38. Rudberg AS, Havervall S, Manberg A, Jernbom Falk A, Aguilera K, Ng H, et al. SARS-CoV-2 exposure, symptoms and seroprevalence in healthcare workers in Sweden. Nat Commun. 2020;11(1):5064.

39. Akyala AI, Awayimbo JR, Elayo MI, Olugbade OT, Akabe EA, Akinyoade A. Severe Acute Respiratory Syndrome Coronavirus 2 (SARS-CoV-2) infection among health care workers in Nasarawa State, Nigeria: implications for infection prevention and control measures. Pan Afr Med J. 2020;37(Suppl 1):21.

40. Augusto JB, Menacho K, Andiapen M, Bowles R, Burton M, Welch S, et al. Healthcare Workers Bioresource: Study outline and baseline characteristics of a prospective healthcare worker cohort to study immune protection and pathogenesis in COVID-19. Wellcome Open Res. 2020;5:179.

41. Barrett ES, Horton DB, Roy J, Gennaro ML, Brooks A, Tischfield J, et al. Prevalence of SARS-CoV-2 infection in previously undiagnosed health care workers in New Jersey, at the onset of the U.S. COVID-19 pandemic. BMC Infect Dis. 2020;20(1).

42. Brant-Zawadzki M, Fridman D, Robinson PA, Zahn M, Chau C, German R, et al. SARS-CoV-2 antibody prevalence in health care workers: Preliminary report of a single center study. PLoS ONE. 2020;15(11 November).

43. Goenka M, Afzalpurkar S, Goenka U, Das SS, Mukherjee M, Jajodia S, et al. Seroprevalence of COVID-19 Amongst Health Care Workers in a Tertiary Care Hospital of a Metropolitan City from India. J Assoc Physicians India. 2020;68(11):14-9.

44. Gras-Valentí P, Chico-Sánchez P, Algado-Sellés N, Gimeno-Gascón MA, Mora-Muriel JG, Jiménez-Sepúlveda NJ, et al. Sero-epidemiological study of SARS-CoV-2 infection among healthcare personnel in a healthcare department. Enferm Infecc Microbiol Clin. 2020.

45. Ko JH, Lee JY, Kim HA, Kang SJ, Baek JY, Park SJ, et al. Serologic Evaluation of Healthcare Workers Caring for COVID-19 Patients in the Republic of Korea. Front Microbiol. 2020;11:587613.

46. Eren E, Çelik İ, Yıldız M, Topaloğlu US, Kılınç-Toker A, Arman-Fırat E, et al. Evaluation of health care workers with COVID-19. Klimik Derg. 2020;33(3):230-4.

47. Mahajan NN, Mathe A, Patojar GA, Bahirat S, Lokhande PD, Rakh V, et al. Prevalence, Clinical Presentations and Treatment Outcomes of COVID-19 among Healthcare Workers at a Dedicated Hospital in India. J Assoc Physicians India. 2020;68(12):16-21.

48. Martin CA, Patel P, Goss C, Jenkins DR, Price A, Barton L, et al. Demographic and occupational determinants of anti-SARS-CoV-2 IgG seropositivity in hospital staff. J Public Health (Oxf). 2020.

49. Rubbi I, Pasquinelli G, Brighenti A, Fanelli M, Gualandi P, Nanni E, et al. Healthcare personnel exposure to COVID - 19: an observational study on quarantined positive workers. Acta Biomed. 2020;91(12-s):e2020012.

50. Abohamr SI, Aldossari MA, Alaklobi FA, Amer HA, Alzarzour SH, Abdelhamid SW, et al. Clinical characteristics and in-hospital outcome of medical staff infected with COVID-19 in Saudi Arabia. A retrospective single-center study. Saudi Med J. 2020;41(12):1336-43.

51. Barallat J, Fernandez-Rivas G, Quirant-Sanchez B, Gonzalez V, Dolade M, Martinez-Caceres E, et al. Seroprevalence of SARS-CoV-2 IgG specific antibodies among healthcare workers in the Northern Metropolitan Area of Barcelona, Spain, after the first pandemic wave. PLoS ONE. 2020;15(12):e0244348.

52. Buonafine CP, Paiatto BNM, Leal FB, de Matos SF, de Morais CO, Guerra GG, et al. High prevalence of SARS-CoV-2 infection among symptomatic healthcare workers in a large university tertiary hospital in Sao Paulo, Brazil. BMC Infect Dis. 2020;20(1):917.

53. Foo DHP, King TL, Lee HC, Santhramogan P, Ganasan V, Fong AYY, et al. COVID-19 testing strategy in response to infection among healthcare workers in a large non-COVID-designated hospital. Hosp Pract (1995). 2020:1-9.

54. Fujita K, Kada S, Kanai O, Hata H, Odagaki T, Satoh-Asahara N, et al. Quantitative SARS-CoV-2 Antibody Screening of Healthcare Workers in the Southern Part of Kyoto City During the COVID-19 Pre-pandemic Period. Front Public Health. 2020;8:595348.

55. Galán MI, Velasco M, Casas ML, Goyanes MJ, Rodríguez-Caravaca G, Losa-García JE, et al. Hospital-Wide SARS-CoV-2 seroprevalence in health care workers in a Spanish teaching hospital. Enferm Infecc Microbiol Clin. 2020.

56. Insúa C, Stedile G, Figueroa V, Hernández C, Svartz A, Ferrero F, et al. Seroprevalence of SARS-CoV-2 antibodies among physicians from a children's hospital. Arch Argent Pediatr. 2020;118(6):381-5.

57. Kim R, Nachman S, Fernandes R, Meyers K, Taylor M, LeBlanc D, et al. Comparison of COVID-19 infections among healthcare workers and non-healthcare workers. PLoS ONE. 2020;15(12):e0241956.

58. Krastinova E, Garrait V, Lecam MT, Coste A, Varon E, Delacroix I, et al. Household transmission and incidence of positive SARS-CoV-2 RT-PCR in symptomatic healthcare workers, clinical course and outcome: a French hospital experience. Occup Environ Med. 2020.

59. Schwartz KL, Achonu C, Buchan SA, Brown KA, Lee B, Whelan M, et al. Epidemiology, clinical characteristics, household transmission, and lethality of severe acute respiratory syndrome coronavirus-2 infection among healthcare workers in Ontario, Canada. PLoS ONE. 2020;15(12):e0244477.

60. Kantele A, Lääveri T, Kareinen L, Pakkanen SH, Blomgren K, Mero S, et al. SARS-CoV-2 infections among healthcare workers at Helsinki University Hospital, Finland, spring 2020: Serosurvey, symptoms and risk factors. Travel Medicine and Infectious Disease. 2021;39.

61. Alshahrani MS, Alnimr A, Alnassri S, Alfarag S, Aljehani Y, Alabdali M. Prevalence of the SARS-CoV-2 Infection Among Post-Quarantine Healthcare Workers. J Multidiscip Healthc. 2020;13:1927-36.

62. Coppeta L, Somma G, Ippoliti L, Ferrari C, D’alessandro I, Pietroiusti A, et al. Contact screening for healthcare workers exposed to patients with covid-19. Int J Environ Res Public Health. 2020;17(23):1-7.

63. Li J, Long X, Zhang Q, Fang X, Li N, Lin Z, et al. Mild manifestations of covid-19 in healthcare workers. PLoS Negl Trop Dis. 2020;14(12):1-5.

64. Mandić-Rajčević S, Masci F, Crespi E, Franchetti S, Longo A, Bollina I, et al. Source and symptoms of COVID-19 among hospital workers in Milan. Occup Med. 2020;70(9):672-9.

65. Lawati AA, Khamis F, Habsi SA, Dalhami KA. Risk of covid-19 infection in healthcare workers exposed during use of non-invasive ventilation in a tertiary care hospital in oman. Oman Medical Journal. 2021;36(2).

66. Escribano Ceruelo E, Espinel Ruíz MA, Ortega López-Peláez M, Fernández Garoz B, Asensio Antón J, Jiménez García R. Seroprevalence of antibodies against SARS-CoV-2 among health care workers in a pediatric monographic hospital in Madrid (Spain). Enfermedades Infecciosas y Microbiologia Clinica. 2021.

67. Goldblatt D, Johnson M, Falup-Pecurariu O, Ivaskeviciene I, Spoulou V, Tamm E, et al. Cross-sectional prevalence of SARS-CoV-2 antibodies in healthcare workers in paediatric facilities in eight countries. J Hosp Infect. 2021;110:60-6.

68. Gupta R, Dwivedi T, Gajendra S, Sahoo B, Gupta S, Vikas H, et al. Seroprevalence of antibodies to SARS-CoV-2 in healthcare workers & implications of infection control practice in India. Indian Journal of Medical Research. 2021;153(1):207-13.

69. Huete-Pérez JA, Cabezas-Robelo C, Páiz-Medina L, Hernández-Álvarez CA, Quant-Durán C, McKerrow JH. First report on prevalence of SARS-CoV-2 infection among health-care workers in Nicaragua. PLoS ONE. 2021;16(1 January).

70. Johnson A, Vincent B, Carson P, Skoy E. Prevalence of SARS-CoV-2 antibodies among North Dakota community pharmacy personnel: A seroprevalence survey. Journal of the American Pharmacists Association. 2021;61(3):e127-e32.

71. Abdelmoniem R, Fouad R, Shawky S, Amer K, Elnagdy T, Hassan WA, et al. SARS-CoV-2 infection among asymptomatic healthcare workers of the emergency department in a tertiary care facility. J Clin Virol. 2021;134.

72. Abdi A, Ahmed AY, Abdulmunim M, Karanja MJ, Solomon A, Muhammad F, et al. Preliminary findings of COVID-19 infection in health workers in Somalia: A reason for concern. Int J Infect Dis. 2021;104:734-6.

73. Nicolás D, Camós-Carreras A, Spencer F, Arenas A, Butori E, Maymó P, et al. A Prospective Cohort of SARS-CoV-2-Infected Health Care Workers: Clinical Characteristics, Outcomes, and Follow-up Strategy. Open Forum Infect Dis. 2021;8(1).

74. Olayanju O, Bamidele O, Edem F, Eseile B, Amoo A, Nwaokenye J, et al. SARS-CoV-2 seropositivity in asymptomatic frontline health workers in Ibadan, Nigeria. Am J Trop Med Hyg. 2021;104(1):91-4.

75. Olmos C, Campaña G, Monreal V, Pidal P, Sanchez N, Airola C, et al. SARS-CoV-2 infection in asymptomatic healthcare workers at a clinic in Chile. PLoS ONE. 2021;16(1 January).

76. Platten M, Cranen R, Peters C, Wisplinghoff H, Nienhaus A, Bach AD, et al. Prevalence of SARS-CoV-2 in employees of a general hospital in Northrhine-Westphalia, Germany. Deutsche Medizinische Wochenschrift. 2021;146(5):E30-E8.

77. Díez-Manglano J, Solís-Marquínez MN, Álvarez García A, Alcalá-Rivera N, Maderuelo Riesco I, Gericó Aseguinolaza M, et al. Healthcare workers hospitalized due to COVID-19 have no higher risk of death than general population. Data from the Spanish SEMI-COVID-19 Registry. PLoS ONE. 2021;16(2):e0247422.

78. Comelli A, Focà E, Sansone E, Tomasi C, Albini E, Quiros-Roldan E, et al. Serological response to sars-cov-2 in health care workers employed in a large tertiary hospital in lombardy, northern italy. Microorg. 2021;9(3):1-13.

79. Jungo S, Moreau N, Mazevet ME, Ejeil AL, Duplan MB, Salmon B, et al. Prevalence and risk indicators of first-wave COVID-19 among oral health-care workers: A French epidemiological survey. PLoS ONE. 2021;16(2 Febuary).

80. Khan MS, Haq I, Qurieshi MA, Majid S, Bhat AA, Qazi TB, et al. SARS-CoV-2 Seroprevalence Among Healthcare Workers by Workplace Exposure Risk in Kashmir, India. Journal of hospital medicine. 2021;16(5):274-81.

81. Lankarani KB, Honarvar B, Omidifar N, Pakdin M, Moghadami M, Shokripour M, et al. Prevalence of anti-sars-cov-2 antibody in hospital staff in double-center setting: A preliminary report of a cohort study from Iran. Shiraz E Medical Journal. 2021;22(3):1-14.

82. Ledda C, Carrasi F, Longombardo MT, Paravizzini G, Rapisarda V. SARS-CoV-2 seroprevalence post-first wave among primary care physicians in catania (Italy). Tropical Medicine and Infectious Disease. 2021;6(1).

83. Lumley SF, O’Donnell D, Stoesser NE, Matthews PC, Howarth A, Hatch SB, et al. Antibody status and incidence of SARS-CoV-2 infection in health care workers. New England Journal of Medicine. 2021;384(6):533-40.

84. Handal N, Whitworth J, Blomfeldt A, Espvik HJ, Lysaker E, Berdal JE, et al. Comparison of SARS-CoV-2 infections in healthcare workers with high and low exposures to Covid-19 patients in a Norwegian University Hospital. Infectious Diseases. 2021;53(6):420-9.

85. Jacob JT, Baker JM, Fridkin SK, Lopman BA, Steinberg JP, Christenson RH, et al. Risk Factors Associated with SARS-CoV-2 Seropositivity among US Health Care Personnel. JAMA Netw Open. 2021;4(3).

86. Nygren D, Norén J, De Marinis Y, Holmberg A, Fraenkel CJ, Rasmussen M. Association between SARS-CoV-2 and exposure risks in health care workers and university employees–a cross-sectional study. Infectious Diseases. 2021;53(6):460-8.

87. Airoldi C, Patrucco F, Milano F, Alessi D, Sarro A, Rossi MA, et al. High seroprevalence of sars-cov-2 among healthcare workers in a north italy hospital. Int J Environ Res Public Health. 2021;18(7).

88. Alkurt G, Murt A, Aydin Z, Tatli O, Agaoglu NB, Irvem A, et al. Seroprevalence of coronavirus disease 2019 (COVID-19) among health care workers from three pandemic hospitals of Turkey. PLoS ONE. 2021;16(3):e0247865.

89. Johnson CC, Coleman CM, Sitarik AR, Leon JE, Tibbetts RJ, Cook BC, et al. SARS-CoV-2 RT-PCR positivity and antibody prevalence among asymptomatic hospital-based health care workers. J Clin Virol. 2021;140.

90. Mostafa A, Kandil S, El-Sayed MH, Girgis S, Hafez H, Yosef M, et al. SARS-CoV-2 seroconversion among 4040 Egyptian healthcare workers in 12 resource-limited healthcare facilities: A prospective cohort study. Int J Infect Dis. 2021;104:534-42.

91. Oliveira MSD, Lobo RD, Detta FP, Vieira-Junior JM, Castro TLDS, Zambelli DB, et al. SARS-Cov-2 seroprevalence and risk factors among health care workers: Estimating the risk of COVID-19 dedicated units. Am J Infect Control. 2021.

92. Özdemir A, Çuha MD, Dizman GT, Alp A, Metan G, Sener B. SARS-CoV-2 seroprevalence among healthcare workers: Retrospective analysis of the data from a university hospital in Turkey. Mikrobiyoloji Bulteni. 2021;55(2):223-32.

93. Purswani MU, Bucciarelli J, Tiburcio J, Yagudayev SM, Connell GH, Omidiran AA, et al. SARS-CoV-2 Seroprevalence Among Healthcare Workers by Job Function and Work Location in a New York Inner-City Hospital. Journal of hospital medicine. 2021;16(5):282-9.

94. Sabetian G, Moghadami M, Hashemizadeh Fard Haghighi L, Shahriarirad R, Fallahi MJ, Asmarian N, et al. COVID-19 infection among healthcare workers: a cross-sectional study in southwest Iran. Virology Journal. 2021;18(1).

95. Abbas S, Raza A, Iftikhar A, Khan A, Khan S, Yusuf MA. Seroprevalence of SARS-CoV-2 Antibodies Among Health Care Personnel at a Health Care System in Pakistan. Asia-Pacific Journal of Public Health. 2021.

96. Colaneri M, Novelli V, Cutti S, Muzzi A, Resani G, Monti MC, et al. The experience of the health care workers of a severely hit SARS-CoV-2 referral Hospital in Italy: incidence, clinical course and modifiable risk factors for COVID-19 infection. Journal of public health (Oxford, England). 2021;43(1):26-34.

97. Kindgen-Milles D, Brandenburger T, Braun JFW, Cleff C, Moussazadeh K, Mrosewski I, et al. Prevalence of SARS-COV-2 positivity in 516 German intensive care and emergency physicians studied by seroprevalence of antibodies National Covid Survey Germany (NAT-COV-SURV). PLoS ONE. 2021;16(4):e0248813.

98. Morris CR, Sullivan P, Mantus G, Sanchez T, Zlotorzynska M, Hanberry B, et al. Prevalence of SARS-CoV-2 antibodies in pediatric healthcare workers. Int J Infect Dis. 2021;105:474-81.

99. Ogutlu A, Karabay O, Erkorkmaz U, Guclu E, Sen S, Aydin A, et al. Novel coronavirus seropositivity and related factors among healthcare workers at a university hospital during the prevaccination period: a cross-sectional study. Ann Clin Microbiol Antimicrob. 2021;20(1):31.

100. Yoshihara T, Ito K, Zaitsu M, Chung E, Aoyagi I, Kaji Y, et al. Sars-cov-2 seroprevalence among healthcare workers in general hospitals and clinics in Japan. Int J Environ Res Public Health. 2021;18(7).

101. Faller E, Wyse A, Barry R, Conlon K, Everard C, Finnegan P, et al. Seroprevalence study of SARS-CoV-2 antibodies in healthcare workers following the first wave of the COVID-19 pandemic in a tertiary-level hospital in the south of Ireland. BMJ Open. 2021;11(6).

102. Kataria Y, Cole M, Duffy E, de la Cena K, Schechter-Perkins EM, Bouton TC, et al. Seroprevalence of SARS-CoV-2 IgG antibodies and risk factors in health care workers at an academic medical center in Boston, Massachusetts. Scientific Reports. 2021;11(1).

103. Ken-Dror G, Wade C, Sharma SS, Irvin-Sellers M, Robin J, Fluck D, et al. SARS-CoV-2 antibody seroprevalence in NHS healthcare workers in a large double-sited UK hospital. Clin Med J R Coll Phys Lond. 2021;21(3).

104. Scohy A, Gruson D, Simon A, Kabamba-Mukadi B, De Greef J, Belkhir L, et al. Seroprevalence of SARS-CoV-2 infection in health care workers of a teaching hospital in Belgium: self-reported occupational and household risk factors for seropositivity. Diagnostic Microbiology and Infectious Disease. 2021;100(4).

105. Hasan T, Pham TN, Nguyen TA, Le HTT, Van Le D, Dang TT, et al. Sero-prevalence of sars-cov-2 antibodies in high-risk populations in vietnam. Int J Environ Res Public Health. 2021;18(12).

106. Fukuda H, Seyama K, Ito K, Ai T, Nojiri S, Hori S, et al. SARS-CoV-2 seroprevalence in healthcare workers at a frontline hospital in Tokyo. Scientific Reports. 2021;11(1).

107. Mortgat L, Verdonck K, Hutse V, Thomas I, Barbezange C, Heyndrickx L, et al. Prevalence and incidence of anti-SARS-CoV-2 antibodies among healthcare workers in Belgian hospitals before vaccination: a prospective cohort study. BMJ Open. 2021;11(6):e050824.

108. Shields AM, Faustini SE, Kristunas CA, Cook AM, Backhouse C, Dunbar L, et al. COVID-19: Seroprevalence and Vaccine Responses in UK Dental Care Professionals. J Dent Res. 2021:220345211020270.
